# Transcriptomic assessment of resistance to effects of an aryl hydrocarbon receptor (AHR) agonist in embryos of Atlantic killifish (*Fundulus heteroclitus*) from a marine Superfund site

**DOI:** 10.1186/1471-2164-12-263

**Published:** 2011-05-24

**Authors:** Marjorie F Oleksiak, Sibel I Karchner, Matthew J Jenny, Diana G Franks, David B Mark Welch, Mark E Hahn

**Affiliations:** 1 Rosenstiel School of Marine and Atmospheric Sciences, University of Miami, 4600 Rickenbacker Causeway, Miami, FL 33149 USA; 2 Biology Department, Woods Hole Oceanographic Institution, Woods Hole, MA 02543, USA; 3 University of Alabama, Department of Biological Sciences, Box 870344, Tuscaloosa, AL 35487-0344 USA; 4 Josephine Bay Paul Center for Comparative Molecular Biology and Evolution, Marine Biological Laboratory, Woods Hole, MA 02568 USA

## Abstract

**Background:**

Populations of Atlantic killifish (*Fundulus heteroclitus*) have evolved resistance to the embryotoxic effects of polychlorinated biphenyls (PCBs) and other halogenated and nonhalogenated aromatic hydrocarbons that act through an aryl hydrocarbon receptor (AHR)-dependent signaling pathway. The resistance is accompanied by reduced sensitivity to induction of cytochrome P450 1A (CYP1A), a widely used biomarker of aromatic hydrocarbon exposure and effect, but whether the reduced sensitivity is specific to CYP1A or reflects a genome-wide reduction in responsiveness to all AHR-mediated changes in gene expression is unknown. We compared gene expression profiles and the response to 3,3',4,4',5-pentachlorobiphenyl (PCB-126) exposure in embryos (5 and 10 dpf) and larvae (15 dpf) from *F. heteroclitus *populations inhabiting the New Bedford Harbor, Massachusetts (NBH) Superfund site (PCB-resistant) and a reference site, Scorton Creek, Massachusetts (SC; PCB-sensitive).

**Results:**

Analysis using a 7,000-gene cDNA array revealed striking differences in responsiveness to PCB-126 between the populations; the differences occur at all three stages examined. There was a sizeable set of PCB-responsive genes in the sensitive SC population, a much smaller set of PCB-responsive genes in NBH fish, and few similarities in PCB-responsive genes between the two populations. Most of the array results were confirmed, and additional PCB-regulated genes identified, by RNA-Seq (deep pyrosequencing).

**Conclusions:**

The results suggest that NBH fish possess a gene regulatory defect that is not specific to one target gene such as CYP1A but rather lies in a regulatory pathway that controls the transcriptional response of multiple genes to PCB exposure. The results are consistent with genome-wide disruption of AHR-dependent signaling in NBH fish.

## Background

Changing environmental conditions provide selective pressures that drive adaptive changes in animal populations [1,2]. Among the many environmental stressors that drive adaptation, the presence of toxic chemicals--naturally derived or anthropogenic--can exert strong effects, in part through their ability to affect the survival of sensitive early developmental stages. Although the acute effects of chemicals are widely studied and adaptation to acute effects of pesticides in invertebrates such as insects is well known, the impact of long-term, multi-generational exposure to chemicals on naturally occurring populations of vertebrate animals is not well understood.

One species that has emerged as a valuable model for investigating evolutionary adaptations to chemical exposure is the Atlantic killifish, *Fundulus heteroclitus*. This estuarine teleost has a long history as a subject for research in environmental biology [3-5], and studies over the past two decades have identified several populations of this species that have evolved tolerance or resistance to toxic chemicals [6,7]. Prominent among these are killifish populations that have developed resistance to toxic polynuclear aromatic hydrocarbons (PAHs) and halogenated aromatic hydrocarbons (HAHs) such as 2,3,7,8-tetrachlorodibenzo-*p*-dioxin (TCDD) and planar polychlorinated biphenyls (PCBs) [8].

Evolved resistance of *F. heteroclitus *to PAHs or HAHs, first noted in Newark, NJ [9,10], has also been described in killifish from the Elizabeth River, VA [11-13], New Bedford Harbor, MA [14,15], and several more moderately contaminated sites in New England [8,16]. At all of these sites, killifish embryos, larvae, and adults are much less sensitive to acute toxicity of HAHs and PAHs as compared to fish from less contaminated reference sites. They also exhibit reduced sensitivity to the induction of cytochrome P450 1A (CYP1A), a widely used marker of altered gene expression in response to these compounds. In fish, mammals, and other vertebrate animals, both the induction of CYP1A and the toxic effects of PAHs and HAHs are controlled by the aryl hydrocarbon receptor (AHR), a ligand-activated, bHLH-PAS protein [17-19]. Thus, the results of these studies on PAH/HAH-resistant killifish suggest that certain AHR-regulated genes--or possibly the AHR pathway generally--have become desensitized in the affected populations.

The New Bedford Harbor (NBH) killifish population, the focus of this study, is resistant to the effects of a variety of AHR ligands, including PAHs, β-naphthoflavone, non-*ortho*-substituted PCBs, 2,3,7,8-tetrachlorodibenzofuran, and TCDD. The resistance to induction of CYP1A is present at all life stages and in all tissues, is heritable, and occurs at the level of mRNA, suggesting a transcriptional effect [8,14,15,20,21]. Recent findings suggest that the resistant phenotype is the result of genetic rather than epigenetic mechanisms [22-25].

Previous studies of reduced sensitivity to altered gene expression in PAH/HAH-resistant killifish, including those in NBH, have focused almost exclusively on induction of CYP1A, as measured by changes in CYP1A mRNA, protein, or activity. The role of CYP1A in the mechanism of PAH and HAH embryotoxicity in fish is not yet clear and is likely to be complex. Some studies have demonstrated that CYP1A is not involved in the mechanism of TCDD toxicity in fish [26], whereas this enzyme appears to play a protective role in PAH toxicity [27-30]. An important unanswered question is whether the reduced sensitivity to gene induction in affected populations is specific to CYP1A or a small subset of AHR target genes or, alternatively, reflects a global (i.e. genome-wide) reduction in responsiveness to all AHR-mediated changes in gene expression. The objective of the present study was to address this question using microarray-based gene expression profiling.

The development of microarray resources for *F. heteroclitus *[31,32] has facilitated the comparison of gene expression profiles in individuals from HAH-sensitive and resistant populations. Studies using a 384-gene metabolic array to survey basal gene expression in brain [33] and liver [34] of untreated adult fish found a small number of changes in fish from polluted sites that could represent either induced or adaptive (evolved) changes. More recently, a second generation array containing 7,000 spots (genes) was generated using cDNA libraries from all 40 developmental stages of *F. heteroclitus *[35]. At the only developmental stage investigated (stage 31, corresponding to approximately ~6 days post-fertilization (dpf)), there were few differences in basal gene expression profiles between polluted and reference sites [35], suggesting that the most important differences in gene expression may only occur under conditions of chemical exposure.

In the present study, we used custom microarrays and deep transcriptome sequencing (RNA-Seq using 454 Life Sciences technology) to examine gene expression profiles in control and PCB-treated killifish embryos and early larvae spawned by fish from NBH (PCB-contaminated site) and Scorton Creek, MA (SC; reference site [15]). We compared expression profiles in embryos and early larvae that had been exposed to vehicle (DMSO) or a potent non-*ortho*-PCB (PCB-126; 3,3',4,4',5-pentachlorobiphenyl) early in development and then sampled at 5, 10, or 15 dpf. The results suggest that resistance to altered gene expression in NBH embryos represents a genome-wide loss of sensitivity.

## Methods

### Chemicals

3,3',4,4',5-Pentachlorobiphenyl (PCB-126) was obtained from Ultra Scientific (Hope, RI). Dimethyl sulfoxide (DMSO) was obtained from Sigma-Aldrich (St. Louis, MO).

### Animals and treatment

**Figure 1:**
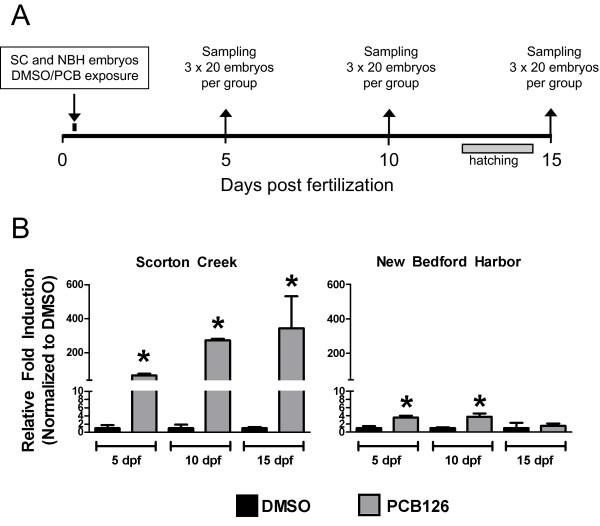
**Experimental design and effect of PCB treatment on expression of CYP1A**. **A**. Experimental design for treatment and sampling. Killifish embryos (4 hpf) were exposed to DMSO or PCB-126 (50 nM) for 1 hr. Sampling was at 5, 10, or 15 dpf. See Methods for additional details. **B**. Expression of CYP1A as measured by real-time RT-PCR. Results (means ± standard deviations of 3 biological replicates) are normalized to expression of beta-actin and expressed relative to the DMSO-treated control in each group. *Statistically significant difference vs. DMSO-treated controls at the same sampling time.

*F. heteroclitus *adults were collected from NBH (New Bedford Harbor, MA; 41°34.0' × 70°54.9') and SC (Scorton Creek, Sandwich, MA; 41°44.0' × 70°23.0') in May and June of 2007, using methods described earlier [15,20]. The fish were transported to the Woods Hole Oceanographic Institution where they were held in 20-gallon continuous flow-through systems in natural seawater for 24 hours until experimentation. For each site, eggs from 8 females (~1100 total) were fertilized using minced testes from 5 males. At 4 hpf, non-fertile eggs were culled and 4- to 8-cell embryos were exposed to vehicle (DMSO; 0.1%) or PCB-126 (50 nM) in filtered seawater (salinity 25 parts per thousand at a density of 65 embryos per 20 ml seawater in glass petri dishes) for 1 hr at 20°C. The chorionic pores of a *Fundulus *egg are ~1.5 μm in diameter [36] and readily allow passage of small chemicals such as PCBs, as confirmed by strong induction of CYP1A (see *Results*). After exposure, the embryos were washed in filtered seawater and incubated at 20°C under a 14-h light, 10-h dark cycle. Embryos were maintained in large petri dishes (150 mm × 60 mm) in filtered seawater (salinity 25 parts per thousand) at a density of one embryo per mL of seawater and water changes were performed every 48 hours. At 5-, 10-, and 15-dpf, embryos were collected as three pools of 20 embryos from each treatment group, flash frozen in liquid nitrogen and stored at -80°C until used for RNA isolation (Figure 1A). Experimental procedures were approved by the Woods Hole Oceanographic Institution's Animal Care and Use Committee (IACUC assurance A3630-01).

### Microarrays

Amplified cDNA sequences for approximately 7,000 genes from *F. heteroclitus *cDNA libraries were spotted onto epoxide slides (Corning) using an inkjet printer (Aj100, ArrayJet, Scotland). Libraries were made from all 40 stages of *F. heteroclitus *development, immediately post-hatch whole larvae, and adult tissues. Each slide contained four spatially separated arrays of ~7,000 spots (genes) including controls. This is an expanded version of previous F. heteroclitus arrays. The current and previous arrays use cDNA probes that have an average length of 1.5 Kb and have a technical variation of less than 5% of the mean (CV < 0.05) [31-35,37-41]. This has allowed us to statistically distinguish less that 1.3 fold differences in expression. All spotted genes were sequenced and represent unique contigs. Thus, even if multiple sequences were annotated identically, they were treated as different genes. Multiple sequences with the same annotation do not contig together because: 1) they really are the same gene, but the sequences do not overlap, 2) they represent duplicate genes with different chromosomal locations, or 3) they share a high similarity (and hence are named based on this similarity) but are not the same gene. We erred on the side of caution and treated every gene-spot as unique.

### Embryo RNA Isolation, Amplification, and Labeling

Total RNA was isolated from pools of embryos (20 embryos per pool) using STAT-60 (Tel-Test), a guanidinium-phenol based nucleic acid isolation method. RNA quality was assessed by gel electrophoresis and prepared for hybridization by one round of amplification (aRNA) using Ambion's Amino Allyl MessageAmp aRNA Kit to form copy template RNA by T7 amplification. Amino-allyl UTP was incorporated into targets during T7 transcription, and resulting amino-allyl aRNA was coupled to Cy3 and Cy5 dyes (GE Healthcare, Piscataway, NJ, USA).

Labeled aRNA samples (2 pmol dye/ul) were hybridized to slides in 10 ul of hybridization buffer (50% formamide buffer, 5× SSPE, 1% sodium dodecyl sulfate, 0.2 mg/ml bovine serum albumin, 1 mg/ml denatured salmon sperm DNA (Sigma), and 1 mg/ml RNAse free poly(A) RNA (Sigma) for 44 hours at 42°C. Slides were prepared for hybridization by blocking in 5% ethanolamine, 100 mM Tris pH 7.8, and 0.1% SDS added just before use for 30 minutes at room temperature, washed for one hour in 4× SSC, 0.1% SDS at 50°C, and then boiled for 2 minutes in distilled water to denature the cDNAs. Resulting 16-bit Tiff Images were quantified using ImaGene^® ^(Biodiscovery, Inc.) spotfinding software. Controls and any gene that did not have at least one individual with a signal greater than the average signal from all herring sperm control spots (non-specific hybridization signal) plus one standard deviation were removed prior to statistical analyses. In total, 6,349 genes were analyzed.

### Quantitative real-time RT-PCR

Two μg total RNA served as template for cDNA synthesis using random hexamers and the Omniscript cDNA Synthesis Kit (Qiagen). Quantitative PCR was performed using the iQ SYBR Green Supermix (Bio-Rad, Hercules, CA) in a MyiQ Single-Color Real-Time PCR Detection system (Bio-Rad). A standard curve was generated by serially diluting plasmids containing a full-length copy of each transcript. Three technical replicates were used for each sample or standard curve dilution. Total molecule numbers were calculated for each sample and normalized by a β-actin correction factor. Changes in expression are reported as changes in fold induction by normalizing molecule numbers to the DMSO control for each time point and respective population (SC or NBH). Real-time PCR primers for CYP1A were: 1A forward primer (5'-CTTTCACAATCCCACACTGCTC-3') and 1A reverse primer (5'-GGTCTTTCCAGAGCTCTG GG -3'). Real-time PCR primers for β-actin were: actin forward primer (5'-TGGAGAAGAGCTAC GAGCTCC-3') and actin reverse primer (5'-CCGCAGGACTCCATTCCGAG-3'). The PCR conditions used here for CYP1A and *β-*actin were: 95°C for 3 min, 95°C for 15 s/62°C for 1 min (40 cycles); followed by a melt curve analysis to ensure that only a single product was amplified.

### Experimental Design for Microarrays

A loop design was used for the microarray hybridizations where each sample is hybridized to two arrays using both Cy3 and Cy5 labeled fluorophores [42,43]. The loop design provides greater statistical power in that it provides twice the amount of data for the same number of microarrays in comparison to a reference design or a design in which only one dye is used [42,44]. Statistical power is particularly important when working with variable natural populations. We used three loops in which each loop consisted of Cy3- and Cy5-labeled embryo aRNAs from 12 samples: one sample from each population-treatment-time combination. Within a population-treatment-time, embryo samples were randomly assigned to one of the three loops. In total, 36 embryo samples were hybridized to 36 microarrays. The loops formed were SC5C→ SC5P → NBH5C→ NBH5P → SC10C→ SC10P→ NBH10C→ NBH10P → SC15C→ SC15P → NBH15C → NBH15P → SC5C (Additional file 1: Fig. S1), where each arrow represents a separate hybridization (array) with the biological sample at the base of the arrow labeled with Cy3 and the biological sample at the head of the arrow labeled with Cy5. SC represents the Scorton Creek, Sandwich, MA population (reference), NBH represents the New Bedford Harbor, MA population, C represents control dose (DMSO), P represents the PCB-126 dose, 5 represents 5-dpf, 10 represents 10-dpf and 15 represents 15-dpf.

### Statistical Analysis of Microarray Data

Log_2 _measures of gene expression were normalized using a linear mixed model in JMP Genomics version 4.1 [45] to remove the effects of dye (fixed effect) and array (random effect) following a joint regional and spatial Lowess transformation in MAANOVA Version 0.98.8 for R to account for both intensity and spatial bias [46].

The model was of the form y_ij _= μ + A_i _+ D_j _+ (AxD)_ij _+ e_ij_, where, y_ij _is the signal from the i^th ^array with dye j, μ is the sample mean, A_i _and D_j _are the overall variation in arrays and dyes (Cy3 and Cy5), (AxD)_ij _is the array × dye interaction and e_ij _is the stochastic error [47,48].

Because we were primarily interested in how PCB treatment differentially affects the sensitive SC and resistant NBH populations rather than differences due to developmental stage, which might confound the analysis of treatment- and population-related differences [41], we analyzed residuals from the above model separately by time (*e.g*., separately for times 5, 10 and 15 dpf). Thus, residuals from the above model were used for gene-by-gene analyses by time of population and treatment effects, using population, treatment and dye as fixed effects, and array as a random effect. The model was r_ijn _= μ + A_i _+ D_j _+ P_n _+ T_k _+ P_n_xT_k _+ e_ijn _where *P_n _*is the *n^th ^*population and *T_k _*is the *k^th ^*treatment (DMSO or PCB-126). For all mixed model analyses, we used a false discovery rate (FDR) p-value of < 0.01 (p < 0.00669) to control for multiple testing [49-51].

Hierarchical clustering of gene expression patterns used Cluster 3.0 for Mac OS × [52] and Java TreeView version 1.0.8 [53]. Correlation analyses used JmpGenomics 4.1 to calculate the Pearson correlation coefficient. Hierarchical clustering of correlation coefficients used Cluster 3.0 for Mac OS × and Matlab version 7.2 [54] for visualization. Significantly correlated genes were graphed using GraphViz version 1.13 [55].

Pairwise comparisons were performed in JMP Genomics version 4.1. Among significantly differentially expressed genes identified by ANOVA, t-statistics were done on least-squares means to identify significant differences between each pair of treatment groups at each sampling time (5-, 10- and 15-dpf).

Results of microarray experiments have been archived in the Gene Expression Omnibus (GEO) database with GEO accession number GSE25245 http://www.ncbi.nlm.nih.gov/geo/query/acc.cgi?acc=GSE25245.

### RNA-Seq

For each treatment group at the 10-dpf time point, equal amounts of total RNA from the three biological replicates were pooled and shipped to Eurofins MWG Operon (Huntsville, AL) for construction of 3'-anchored cDNA libraries and generation of ESTs by pyrosequencing by 454 technology [56]. PolyA^+ ^RNA was purified and a non-normalized library with unique barcodes was prepared from each of four samples (SC-DMSO, SC-PCB, NBH-DMSO, and NBH-PCB). An oligo-dT primer was used for first-strand synthesis and the second strand was random-primed. Following size fractionation to 500-600 base pairs, the libraries were combined and sequenced on a single region of a GS-FLX using Titanium chemistry. The average read lengths were about 330 bases (mode 390) after the removal of key and tag sequences. The total number of reads from each library ranged between 130,000-175,000.

Additional information on the *Fundulus *developmental transcriptome was obtained by deep sequencing of shotgun cDNA libraries prepared from SC and NBH embryos and larvae collected daily from days 1-15 of development. Equal amounts of total RNA from each sampling stage were pooled to capture as much of the killifish embryo transcriptome as possible. PolyA^+ ^RNA was isolated using the MicroPoly(A)Purist kit (Ambion, Austin, TX). Separate developmental libraries were made from SC and NBH fish following instructions for the GS FLX Titanium Rapid Library Preparation kit (Roche, Indianapolis, IN). Briefly, polyA^+ ^RNA was fragmented with a ZnCl_2 _solution prior to first-strand cDNA synthesis with random primers. After the second-strand synthesis, multiplex adaptors were ligated and small fragments were removed. The quantification, emPCR amplification and 454 pyrosequencing of the libraries were performed on a GS-FLX instrument at the Josephine Bay Paul Center of the Marine Biological Laboratory (Woods Hole, MA); the two libraries were sequenced on separate regions of a single run. The number of reads obtained from the SC and NBH embryo libraries were 756,803 and 781,203 respectively. The average read length was about 425 bases (mode 480) after removal of multiplex adaptor sequences. The data was assembled using Newbler, version 2.5 (Roche, Indianapolis, IN).

ESTs (from the treatment-specific, 3'-anchored libraries) were used to obtain an independent measure of gene expression for each of the genes found by microarray to be significantly altered by PCB treatment. The probe sequences for the 40 significant hits on the microarray from the 10-dpf data set were used to search a database of the raw reads from each EST library using BlastStation-Local (version 1.4 [57]). The matching reads from each library were determined and the sequences extracted; the e-value cut-off to be considered a match was approximately 1x10^-20^, and the percent identity limit was set as 90% or higher. All the matching reads for a given probe were combined and assembled into contigs using the CAP3 Sequence Assembly Program [58]. This resulted in the extension of most of the probe sequences for which there were 454 reads. The blast searches against the four EST databases were repeated with the extended probe sequences using the same significance parameters as before. These data are presented as the number of 454 reads corresponding to each microarray probe, for each treatment group at 10-dpf.

## Results

### Effects of PCB-126 on CYP1A expression and embryonic development in SC and NBH embryos

To confirm the effectiveness of the PCB exposure and begin to assess altered gene expression in embryos and larvae from the two populations, we measured CYP1A expression by real-time RT-PCR. Embryos and larvae from SC were highly responsive, showing induction of CYP1A at all three time points, with fold-induction values ranging from 68- to 345-fold (Figure 1B). By contrast, NBH fish were essentially refractory to CYP1A induction (1.5- to 3.8-fold change, PCB vs. DMSO).

*Fundulus *embryos from SC and NBH displayed normal growth during the first five days of development in both the DMSO and PCB treatments. The lack of effect at this time is consistent with the delay in toxic response of embryos to TCDD or PCB-126 seen in other fish [59,60]. However, by 10 dpf the PCB-treated SC embryos began to develop signs of developmental delay and displayed the characteristic pericardial edema phenotype consistent with TCDD- or PCB-induced developmental toxicity. In contrast to the PCB-treated SC embryos, the DMSO-treated SC embryos and the DMSO and PCB-treated NBH embryos developed normally. At 15 dpf, the DMSO-treated SC embryos and both treatments of NBH embryos had hatched and exhibited no signs of developmental abnormalities, whereas the 15 dpf PCB-treated SC embryos were unhatched and displayed severe pericardial edema and vascular hemorrhaging, as well as moderate developmental delays.

Together, the results from analysis of CYP1A expression and embryotoxicity show that the exposure was effective and that the NBH fish have retained the PCB-resistant phenotype that was first described more than a decade ago [8,14,15].

### Gene expression profiles

**Figure 2:**
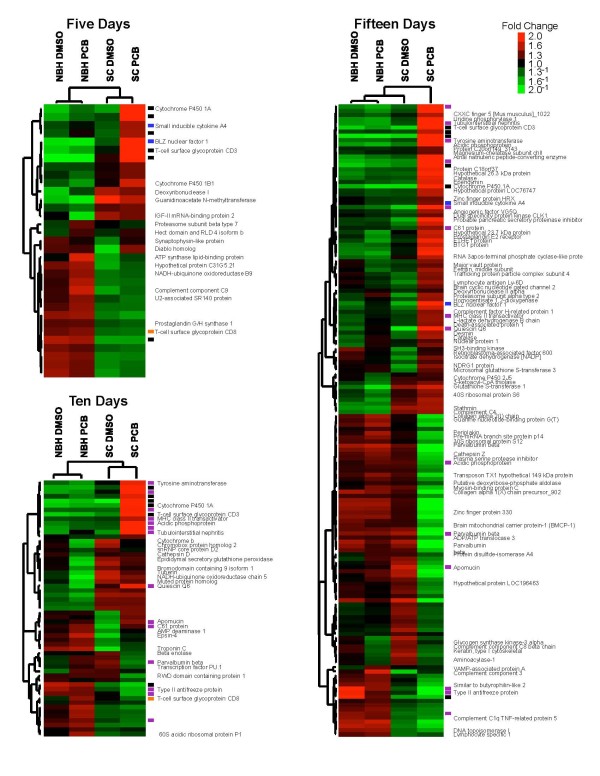
**Heat map illustrating results of hierarchical clustering of the genes whose expression is significantly different due to population, treatment and population-by-treatment interactions at 5, 10, and 15 days post-fertilization**. Clusters of genes with similar expression patterns are shown on the left (gene tree). Red indicates high expression levels and green represents low expression levels. Black rectangles denote genes significantly differently expressed at 5, 10, and 15 days post-fertilization. Orange rectangles denote genes significantly differently expressed at 5 and 10 days post-fertilization. Blue rectangles denote genes significantly differently expressed at 5 and 15 days post-fertilization. Purple rectangles denote genes significantly differently expressed at 10 and 15 days post-fertilization. Unlabeled rectangles represent unannotated genes.

Gene expression data were analyzed to identify differences associated with population, treatment, or the combination. At a FDR p-value of 0.01, approximately 3.8% (242/6349) of genes were significantly differentially expressed. Thirty-three genes (0.5%) were significantly differentially expressed at 5 dpf (Figure 2; Additional file 2: Table S1; Additional file 3: Fig. S2). Twenty-six of these genes had a significant population effect, nine had a significant treatment effect, and nine had a significant population-by-treatment effect. Fifty-seven genes (0.9%) were significantly differentially expressed at 10 dpf. Twenty-seven of these genes had a significant population effect, 23 had a significant treatment effect, and 24 had a significant population-by-treatment effect. One-hundred and fifty-two genes (2.4%) were significantly differentially expressed at 15 dpf. Forty of these genes had a significant population effect, 69 had a significant treatment effect, and 105 had a significant population-by-treatment effect.

### Correlation analyses

**Figure 3:**
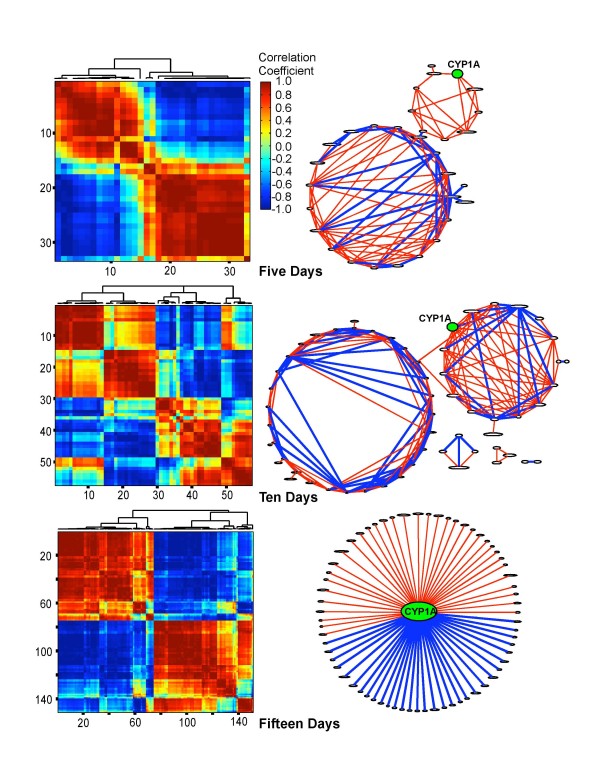
**Correlations and network interactions of genes whose expression is significantly different due to population, treatment and population-by-treatment interactions at 5, 10 and 15 days post-fertilization**. Correlation coefficients > 0.95 and < -0.95 are significant at p < 0.01. Red indicates positive correlations and blue indicates negative correlations. In the gene networks, red lines connect genes with significant positive correlations and blue lines connect genes with significant negative correlations (p < 0.01). Green ovals denote CYP1A in the different networks. Because of the complexity of the networks at 15 days post-fertilization, only the network of genes with significant interactions with CYP1A is shown.

Among the 33 genes significantly differentially expressed at 5 dpf, 22.3% had a significant correlation coefficient (> 0.94 or < -0.94, p < 0.01): 17.2% were significantly positively correlated and 5.1% were significantly negatively correlated (Figure 3). At 10 dpf, fewer genes had a significant correlation coefficient (13.5%): 9.2% were positively correlated and 4.3% were negatively correlated. At 15 dpf, 22.4% had a significant correlation, a percentage similar to that of the 5 day exposed embryos. However, unlike either the 5- or 10-dpf embryos, for which more positive correlations were observed, in the 15-dpf embryos the percentages of positively and negatively correlated genes were similar (10.8% negatively correlated *versus *11.6% positively correlated). CYP1A correlations reflected the overall correlation patterns. At 5 dpf, CYP1A was significantly positively correlated with four genes. At 10 dpf, CYP1A was significantly positively correlated with ten genes and negatively correlated with one gene, and at 15 dpf, CYP1A was positively correlated with 35 genes and negatively correlated with 31 genes (Figure 3).

**Figure 4:**
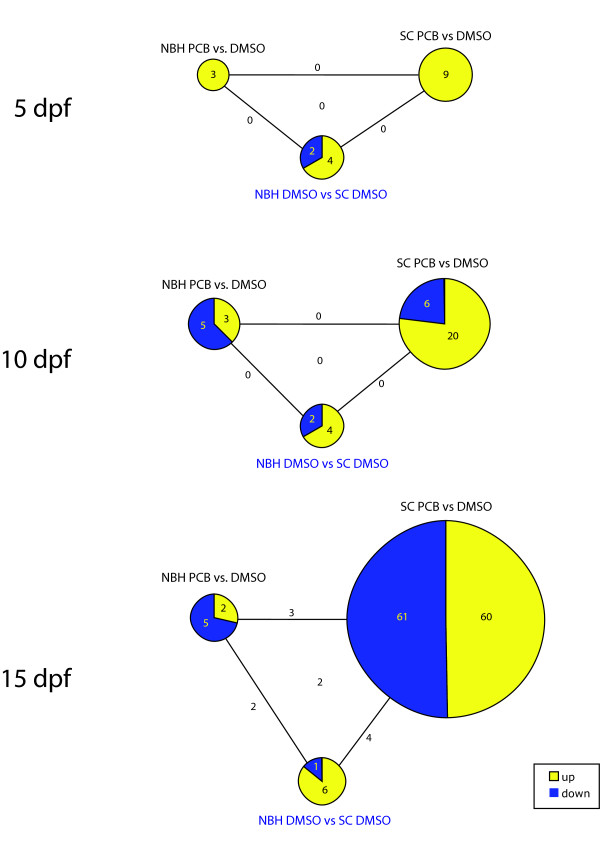
**Number of genes exhibiting statistically significant differences in expression in pairwise comparisons of NBH PCB vs NBH DMSO, SC PCB vs SC DMSO, and NBH DMSO vs SC DMSO**. The size of the circles reflects the number of genes in each group. Numbers on lines indicate number of genes shared between the groups. See Table 1 and Additional file 4: Table S2 for gene lists.

To directly compare the responses occurring in SC and NBH fish, we performed pairwise comparisons, focusing on the genes that exhibited significantly altered expression as a result of PCB exposure in each population, at each time. The results are summarized in Figure 4 and the genes are listed in Table 1 and Additional file 4: Table S2. At 5 dpf, nine genes were differentially expressed in the PCB-treated SC embryos as compared to the DMSO-treated SC embryos; all nine genes were induced. In the NBH embryos, only three genes were altered by PCB treatment (all induced). The genes induced in NBH embryos were distinct from those induced in SC embryos. In addition to having more genes that responded to PCB exposure, the SC embryos displayed a greater magnitude of response, with an average 3-fold change as compared to the 1.5-fold average change in NBH embryos (Additional file 5: Table S3).

**Table 1:** Differential gene expression and PCB inducibility in pairwise comparisons of NBH and SC embryos at 5 and 10 dpf.

Gene	NBH PCB/NBH DMSO	SC PCB/SC DMSO	NBH DMSO/SC DMSO
**5 days post fertilization**			

basic leucine zipper nuclear factor 1 [Mus musculus]_603	-1.03	**5.09**^c^	-1.52
Cytochrome P450 1A1 (EC 1.14.14.1) (CYP1A)_1115	1.29	**4.82**^b, c^	-1.12
*UnAn_29411_6378 (CYP1B1)	-1.03	**4.61**^b, c^	-2.51
T-cell surface glycoprotein CD3 delta chain precursor (T-cell receptor T3 delta chain)_3770	1.14	**2.92**^b, c^	-1.64
*UnAn_27910_5543 (CYP1B1)	1.22	**2.54**^b, c^	-1.32
Diablo homolog, mitochondrial precursor (Second mitochondria-derived activator of caspase) (Smac protein) (Direct IAP binding protein with low pI)_1191	-1.03	**2.40**	2.14
UnAn_21996_4295	1.28	**1.84**^b, c^	1.02
Small inducible cytokine A4 homolog precursor (Macrophage inflammatory protein 1-beta homolog)_3581	1.13	**1.78**^c^	1.03
Synaptophysin-like protein (Pantophysin)_3753	-1.15	**1.43**	1.64
ATP synthase lipid-binding protein, mitochondrial precursor (EC 3.6.3.14) (ATP synthase proteolipid P2) (ATPase protein 9) (ATPase subunit C)_560	**1.79**	1.31	1.47
Hypothetical protein C31G5.21 in chromosome I_1963	1.12	1.27	**1.98**
hect domain and RLD 4 isoform b [Homo sapiens]_1806	**1.33**	1.21	-1.06
UnAn_26723_4896	-1.14	1.14	**2.12**
UnAn_23246_4614	-1.09	1.03	**1.96**
UnAn_22726_4401	1.03	-1.10	**1.88**
Proteasome subunit beta type 7 precursor (EC 3.4.25.1) (Proteasome subunit Z) (Macropain chain Z) (Multicatalytic endopeptidase complex chain Z)_3109	**1.41**	-1.10	-1.14
Deoxyribonuclease-1 precursor (EC 3.1.21.1) (Deoxyribonuclease I) (DNase I)_1181	1.48	-1.14	**-2.57**
Guanidinoacetate N-methyltransferase (EC 2.1.1.2)_1754	-1.16	-1.25	**-3.28**

**10 days post fertilization**			

*UnAn_29411_6378 (CYP1B1)	1.09	**11.78**^a, c^	1.12
Cytochrome P450 1A1 (EC 1.14.14.1) (CYP1A)_1115	1.10	**6.10**^a, c^	-1.13
T-cell surface glycoprotein CD3 delta chain precursor (T-cell receptor T3 delta chain)_3770	1.07	**5.47**^a, c^	-1.25
*UnAn_27910_5543 (CYP1B1)	-1.02	**3.36**^a, c^	-1.29
Tubulointerstitial nephritis antigen-like precursor (Androgen-regulated gene protein 1) (Adrenocortical zonation factor 1) (AZ-1) (Tubulointersititial nephritis antigen-related protein) (TARP)_4042	1.49	**3.00**^c^	-1.03
**UnAn_29159_6257 (apolipoprotein E)	1.18	**2.66**^c^	-1.20
**UnAn_28041_5632 (apolipoprotein E)	1.02	**2.43**^c^	-1.01
Acidic phosphoprotein precursor (50 kDa antigen)_277	1.11	**2.37**^c^	-1.15
**UnAn_23610r_4780 (apolipoprotein E)	1.06	**2.30**^c^	-1.45
UnAn_21996_4295	-1.15	**2.09**^a, c^	-1.01
Apomucin (Mucin core protein) (Fragment)_492	1.16	**2.02**^c^	1.45
C61 protein [Mus musculus]_684	1.22	**1.99**^c^	1.84
WAP four-disulfide core domain protein 3 precursor (Putative protease inhibitor WAP14)_6790	1.02	**1.96**	1.59
Tyrosine aminotransferase (EC 2.6.1.5) (L-tyrosine:2-oxoglutarate aminotransferase) (TAT)_4064	-1.14	**1.90**^c^	-1.24
MHC class II transactivator (CIITA)_2363	1.03	**1.76**^c^	-1.06
UnAn_23121_4564	-1.09	**1.75**	1.54
Troponin C, slow skeletal and cardiac muscles (TN-C)_3995	1.08	1.63	**2.68**
Epsin-4 (Epsin-related protein) (EpsinR) (Enthoprotin)_1384	1.38	**1.62**	2.03
UnAn_27985_5592	-1.10	**1.44**	1.13
AMP deaminase 1 (EC 3.5.4.6) (Myoadenylate deaminase) (AMP deaminase isoform M)_431	1.27	**1.43**	1.49
UnAn_29343_6349	1.20	**1.31**	1.48
UnAn_29849_6655	1.11	1.24	**1.67**
Cathepsin D precursor (EC 3.4.23.5)_755	**-1.45**	1.04	1.01
UnAn_22785_4432	-1.24	-1.01	**-1.84**
UnAn_20648_4152	**1.32**	-1.05	1.01
Mitogen-activated protein kinase kinase 1 interacting protein 1 (MEK binding partner 1) (Mp1)_2424	-1.10	-1.07	**-1.51**
Epididymal secretory glutathione peroxidase precursor (EC 1.11.1.9) (Epididymis-specific glutathione peroxidase-like protein) (EGLP)_1378	**-1.73**	-1.15	-1.32
Muted protein homolog_2461	**-1.60**	-1.16	-1.50
UnAn_22879_4456	**1.56**	-1.20	-1.51
UnAn_22354_4332	-1.09	-1.28	**2.13**^c^
Type II antifreeze protein precursor (AFP)_4059	-1.12	-1.30	**1.72**^c^
T-cell surface glycoprotein CD8 beta chain precursor (CD8 antigen 37 kDa chain) (OX-8 membrane antigen)_3771	**1.51**	-1.32	-1.12
UnAn_20957_4180	**-1.84**	-1.32	-1.48
Chromobox protein homolog 2 (Modifier 3 protein) (M33)_827	**-1.65**	-1.47	-1.36
Beta enolase (EC 4.2.1.11) (2-phospho-D-glycerate hydro-lyase) (Muscle-specific enolase) (MSE) (Skeletal muscle enolase) (Enolase 3)_620	-1.00	**-1.53**	-1.29
UnAn_27466_5284	1.30	**-1.54**	-1.33
Transcription factor PU.1_3890	1.19	**-1.55**	-1.36
UnAn_22873_4452	-1.00	**-1.55**^c^	1.30
RWD domain containing protein 1 (Small androgen receptor-interacting protein)_3458	1.05	**-1.73**	-1.05
Parvalbumin beta_2778	1.30	**-1.80**^c^	-1.49

Genes with significant differences in pairwise comparisons of gene expression at 5 and 10 dpf are included. (For 15-dpf data, see Additional file 4**: Table S2**.) Gene expression ratios are indicated. A gene with a positive fold-difference is more highly expressed in the population/treatment listed first, and a gene with a negative fold-difference is more highly expressed in the population/treatment listed last. Genes are listed in order of ratios in the reference population (SC) comparison with tolerant population (SC). Ratios with significant p-values are in **bold**. See **Table S1 (**Additional file 2) for a list of all genes significant in the ANOVA analysis. NBH: New Bedford Harbor; SC: Scorton Creek; PCB: PCB-126; DMSO: dimethylsulfoxide. Unannotated genes are denoted by UnAn and a unique number. Some of the unannotated probes were subsequently annotated after extension using the 454 database; see **Table S4 (**Additional file 6) for details.

*These two probes represent the same transcript, which has been annotated as cytochrome P450 1B1 (CYP1B1) using data from 454 libraries.

** These three probes represent the same transcript, which has been annotated as apolipoprotein E using data from 454 libraries.

^a^Also differentially expressed at 5 dpf.

^b^Also differentially expressed at 10 dpf.

^c^Also differentially expressed at 15 dpf.

In SC embryos sampled at 10 dpf, there were twenty-six genes with PCB-altered expression (20 up-regulated and 6 down-regulated versus DMSO-treated embryos), whereas in the NBH embryos only eight genes were affected (3 up and 5 down). As in the 5-dpf embryos, there was no overlap in the sets of genes with PCB-altered expression in the 10-dpf SC and NBH embryos, and the SC embryos exhibited a greater magnitude of change (2.6-fold versus 1.6-fold).

At 15 dpf, there was a dramatic increase in the number of genes affected by PCB exposure in SC larvae, with 121 significant responses (60 up- and 61 down-regulated) (Figure 4; Additional file 4: Table S2). In contrast, only seven genes were altered by PCB in NBH larvae at this time (2 up and 5 down). Three genes were significantly down-regulated in both populations: complement C8 beta chain precursor, type II antifreeze protein precursor, and an unannotated gene (UnAn_22873). As seen at the other times, the average magnitude of change was greater in SC larvae as compared to NBH larvae (2.3-fold vs. 1.4-fold).

To examine genes that may exhibit differences in basal expression between NBH and SC embryos, we performed a pairwise comparison of the control (DMSO-exposed) fish from each site at each time point. The number of genes exhibiting differential expression in NBH and SC fish at 5, 10, and 15 dpf was 6, 6, and 7, respectively; most were more highly expressed in NBH fish (Figure 4; Table 1; Additional file 4: Table S2). There was little overlap among sampling times, except for two genes that were more highly expressed in NBH fish at both the 10- and 15-dpf sampling times: type II antifreeze protein precursor and an unannotated gene (UnAn_22354).

### Deep sequencing

To obtain an independent assessment of genes with PCB-altered expression in the two populations, we examined expression in the 10-dpf samples using RNA-Seq. The probe sequences for the 40 significant hits on the microarray from the 10-dpf data set were used to search a database of ESTs from each library, as described in *Methods*. Most of the microarray probes (27/40) were represented by reads in the EST libraries. Blast searches against the shotgun cDNA assemblies from SC and NBH embryos using the significant array probes resulted in further extension of some of the probe sequences. In addition, we detected matching reads or contigs in the shotgun library for 8 of 13 probes for which there were no matching EST reads. The extended probe sequences were used to search the EST databases and matches were found for two probes for which previously there had been no matching reads. The number of reads for all 40 probes is listed in Table 2.

**Table 2:** Differential gene expression in 10-dpf NBH and SC embryos as measured by RNA-Seq.

Gene	SC-DMSO	SC-PCB	NBH-DMSO	NBH-PCB
UnAn_29411_6378* (CYP1B1) #	0	66	0	0
Cytochrome P450 1A1 (EC 1.14.14.1) (CYPIA1)_1115 #	0	4987	0	3
T-cell surface glycoprotein CD3 delta chain precursor (T-cell receptor T3 delta chain)_3770 #	0	0	0	0
UnAn_27910_5543* (CYP1B1) #	0	66	0	0
Tubulointerstitial nephritis antigen-like precursor (Androgen-regulated gene protein 1) (Adrenocortical zonation factor 1) (AZ-1) (Tubulointersititial nephritis antigen-related protein) (TARP)_4042 #	1	0	3	6
UnAn_29159_6257** (apolipoprotein E) #	41	177	52	37
UnAn_28041_5632** (apolipoprotein E) #	41	177	52	37
Acidic phosphoprotein precursor (50 kDa antigen)_277 #	0	0	0	0
UnAn_23610r_4780** (apolipoprotein E) #	41	177	52	37
UnAn_21996_4295 #	0	0	0	0
Apomucin (Mucin core protein) (Fragment)_492 #	18	31	8	10
C61 protein [Mus musculus]_684 #	0	0	0	2
WAP four-disulfide core domain protein 3 precursor (Putative protease inhibitor WAP14)_6790 #	0	0	0	0
Tyrosine aminotransferase (EC 2.6.1.5) (L-tyrosine:2-oxoglutarate aminotransferase) (TAT)_4064 #	0	0	0	0
MHC class II transactivator (CIITA)_2363 #	0	0	0	0
UnAn_23121_4564 #	2	0	3	0
Troponin C, slow skeletal and cardiac muscles (TN-C)_3995	13	26	22	14
Epsin-4 (Epsin-related protein) (EpsinR) (Enthoprotin)_1384 #	0	0	0	0
UnAn_27985_5592 #	19	10	14	11
AMP deaminase 1 (EC 3.5.4.6) (Myoadenylate deaminase) (AMP deaminase isoform M)_431 #	0	0	0	0
UnAn_29343_6349 #	134	179	92	137
UnAn_29849_6655	2	0	0	0
Cathepsin D precursor (EC 3.4.23.5)_755	0	0	1	0
UnAn_22785_4432	129	103	77	135
UnAn_20648_4152	5	0	9	8
Mitogen-activated protein kinase kinase 1 interacting protein 1 (MEK binding partner 1) (Mp1)_2424	11	7	5	13
Epididymal secretory glutathione peroxidase precursor (EC 1.11.1.9) (Epididymis-specific glutathione peroxidase-like protein) (EGLP)_1378	3	4	0	1
Muted protein homolog_2461	22	5	4	7
UnAn_22879_4456	50	35	42	32
UnAn_22354_4332	12	22	32	25
Type II antifreeze protein precursor (AFP)_4059	2	1	2	0
T-cell surface glycoprotein CD8 beta chain precursor (CD8 antigen 37 kDa chain) (OX-8 membrane antigen)_3771	11	9	13	12
UnAn_20957_4180	0	0	1	2
Chromobox protein homolog 2 (Modifier 3 protein) (M33)_827	0	0	0	0
Beta enolase (EC 4.2.1.11) (2-phospho-D-glycerate hydro-lyase) (Muscle-specific enolase) (MSE) (Skeletal muscle enolase) (Enolase 3)_620	18	15	18	20
UnAn_27466_5284	12	12	14	19
Transcription factor PU.1_3890	0	0	0	0
UnAn_22873_4452	0	0	0	0
RWD domain containing protein 1 (Small androgen receptor-interacting protein)_3458	15	9	10	1
Parvalbumin beta_2778	49	44	40	60

The probe sequences representing genes with significant differences in pairwise comparisons in the 10-dpf samples (see Table 1) were used in blast searches against a database of the reads from each EST library using BlastStation-Local (version 1.4). The number of reads matching each probe sequence is indicated. The e-value cut-off was approximately 1x10^-20^, and the percent identity limit was set as 90% or higher. NBH: New Bedford Harbor; SC: Scorton Creek; PCB: PCB-126; DMSO: dimethylsulfoxide. Unannotated genes are denoted by UnAn and a unique number. The probes labeled with asterisks (* or **) belong to the same transcript, and therefore share the same 454 read counts. Some of the unannotated probes were subsequently annotated after extension using the 454 database; see Table S4 (Additional file 6) for details. Probes labeled with "#" are those exhibiting significant induction by PCB in SC embryos but not NBH embryos in the microarray analysis (see Table 1).

Among the 20 probes that showed significant PCB-induction in SC, but not NBH fish in the microarray analyses, 12 genes had corresponding RNA-Seq data. Three of the twelve genes had very few reads in the libraries, and of the nine remaining genes, seven (78%) showed the same trend as the microarray data. For the two genes that did not show the same trend as the microarray data, one (UnAn_29343) showed induction in SC fish that was similar to that measured by microarray but also showed induction in NBH fish, and the other (UnAn_27985) showed a reduction in read number (rather than induction) in PCB-treated SC embryos (Table 2). By extending the probe sequences using the shotgun cDNA assemblies, we were able to annotate eleven previously unannotated probes from the 10 dpf significant hits list (Additional file 6: Table S4). The extension of the probe sequences also revealed that some of the probes represent fragments from the same transcript. For example, the three probes UnAn_23610r, UnAn_28041, and UnAn_29159 belong to the same transcript (apolipoprotein E), as do the two probes UnAn_27910 and UnAn_29411 (part of the 3'-UTR of the CYP1B1 transcript). This is supported by the finding that these probes exhibited similar results in the array (2.3-, 2.43-, and 2.66-fold for the the apolipoprotein E probes, 3.36- and 11.78-fold for the CYP1B1 probes).

Further analysis of the RNA-Seq and cDNA data for sequences not represented on the array revealed additional genes responsive to PCB (induced or repressed) in both populations. Analysis of these data is ongoing; results will be reported in a subsequent manuscript.

## Discussion

We compared gene expression profiles and the response to PCB exposure in embryos (5 and 10 dpf) and larvae (15 dpf) from two *F. heteroclitus *populations: SC and NBH. The SC population is sensitive to the typical early life stage toxicities associated with exposures to halogenated aromatic hydrocarbons including edema, circulatory failure, craniofacial malformations, and death. In contrast, the NBH population is resistant to typical HAH toxicities [8,14,15]. We examined gene expression using a 7,000-gene, cDNA array. Our results reveal striking differences in responsiveness to PCB between the populations; the differences occur at all three stages examined. There was a sizeable set of PCB-responsive genes in the sensitive SC population, a much smaller set of PCB-responsive genes in NBH fish, and few similarities in PCB-responsive genes between the two populations. The results suggest that the attenuated response to PCB in NBH fish extends beyond the typical responses such as induction of CYP1A, and that entire response pathways are regulated differently in this PCB-tolerant population.

### Population and Treatment effects

Population effects dominated the significant changes in gene expression in day 5 embryos. Twenty-six genes (79% of 33 significant genes) have a significant population effect; twenty of these genes (61%) were significant only due to population (*i.e*., they did not have a significant treatment or population-by-treatment effect, Additional file 2: Table S1). These genes drive the hierarchical clustering pattern in 5-dpf embryos (Figure 2). The differences in gene expression between populations are unlikely to be physiologically induced because embryos were generated within a two-week period and cultured in a constant, common environment. However, parental differences that may affect offspring (*e.g*., health, age, diet) cannot be ruled out, and some of the apparent differences in gene regulation could be the result of subtle differences in developmental rates between the SC and NBH populations [41] or differential sensitivity to changes in developmental rates in response to PCB. Genes with a significant population effect also drove the clustering in 10-dpf embryos but were less dominant (27 genes, 47% of 57 significant genes); treatment and population-by-treatment effects were nearly as strong (23 genes each) (Additional file 2: Table S1). Because we only compared embryos from two populations, we cannot determine whether the population differences are the result of genetic drift or selection. Population or strain differences in gene expression unrelated to sensitivity to PCBs or dioxins have been observed in *Fundulus *embryos [35], Atlantic tomcod embryos [61], and strains of rats [62,63].

In contrast to what was seen in 5 and 10 dpf embryos, population-by-treatment effects drove the clustering pattern in 15 dpf embryos, and the PCB-treated SC embryos clustered separately from the other groups (Figure 2). While only 26% of the genes (40 of 151 genes) had a significant population effect in these day 15 embryos, 46% (69 genes) had a significant treatment effect and 70% (105 genes) had a significant population-by-treatment effect. This dramatic increase in the interaction between population and treatment likely reflects the progressive embryotoxicity that was being experienced by the SC embryos, but not the NBH embryos, starting at about 10-dpf. Similar results involving large strain-specific differences in treatment responses linked to differential susceptibility to toxicity were described recently in rats exposed to TCDD [64]. That we did not see even more dramatic changes in gene expression in the 15-dpf embryos may reflect the relatively stringent significance criteria used (FDR of 0.01).

### Comparison of microarray and deep sequencing results

For the 10 dpf samples, we used deep sequencing of 3'-anchored EST libraries (RNA-Seq) to provide an independent assessment of altered gene expression. Of the 40 genes exhibiting significantly altered expression in the pairwise comparisons, approximately one fourth were not found in the EST libraries (Table 2). Some of these may represent relatively rare transcripts, for which our depth of sequencing (130,000-175,000 reads per library) was not sufficient. In fact, the increased sequencing depth of the shotgun cDNA libraries provided contigs matching more than half of the probes for which there were no reads in the EST libraries. The location of the array probe along the transcript could also be a factor for probes lacking matching reads. Because the ESTs are 3'-anchored cDNA fragments, they would not have matches to probes representing other parts of the transcript. Additional analysis of the EST and cDNA assemblies, leading to a more complete representation of the *Fundulus *transcriptome, is ongoing and will be reported separately.

### CYP1A induction and other population-specific responses: implications for AHR-dependent signaling

A well-studied gene with a significant population-by-treatment effect at all three sampling times is CYP1A. Induction of CYP1A expression is widely used as a biomarker of exposure to contaminants including PAHs and HAHs and is a readily measured endpoint in studies of the mechanisms of AHR signaling [65]. CYP1A shows reduced sensitivity to induction in multiple populations of *Fundulus *from contaminated sites, including NBH [8,14,15,20], and poor responsiveness to CYP1A induction is a good predictor of resistance to PAH- or HAH-induced embryotoxicity [8]. Thus, CYP1A can serve as a benchmark with which to compare other PCB-induced changes and can be used to identify other potentially AHR-regulated genes in SC fish that appear to be regulated differently in the NBH fish.

Several genes show the same pattern as CYP1A in all pairwise comparisons of treatment groups, i.e. SC fish treated with PCB are significantly different from the other three groups (SC fish treated with DMSO, NBH fish treated with PCB or DMSO) and there are no significant differences in other pairwise comparisons. At 5 dpf, five genes (15% of 33 significant genes) show this pattern, at 10 dpf, twelve genes (21% of 57 significant genes) and at 15 dpf, fifty-six genes (38% of 151 significant genes) show this pattern. Most of these genes (including 3/5 at 5 dpf and 10/12 at 10 dpf) also show a significant positive correlation with CYP1A in the correlation analysis (Figure 3).

The genes showing a CYP1A-like pattern of induction or correlation to CYP1A can be considered candidates for genes that exhibit AHR-dependent regulation and, like CYP1A (Figure 1b) and AHRR ([66] and unpublished results in embryos), display a suppressed induction response in NBH fish. Some of these include basic leucine zipper NF-1, T-cell surface glycoprotein CD3 delta chain, MHC class II transactivator (CIITA), apolipoprotein E, and CYP1B1. Two additional genes that displayed this pattern (cytochrome b5 and S100 calcium-binding protein A11) were identified from deep sequencing data (see below). The precise role of AHRs in regulating each of these genes will require further experimentation.

Additional insight concerning possible differences between SC and NBH populations in the functioning of the AHR pathway is provided by the two key pairwise comparisons that reveal the sets of genes altered by PCB in each population (Figure 4). There was a progressive increase in the number of genes with significantly altered expression over time in the SC population (from 9 genes at 5 dpf to 121 at 15 dpf) but not in the NBH population (from 3 to 7 genes over this same time interval). The small number of PCB-altered genes is unlikely to be related to the onset of zygotic transcription, which occurs much earlier in killifish (typically by 6 hr post fertilization [41]). While the initial PCB-induced changes (5 dpf) all involved up-regulation of gene expression, half of the changes at 15 dpf involved down-regulation. Our 5 dpf results are consistent with results from other systems, where inductive effects on gene expression usually dominate over repressive effects after acute exposure to AHR agonists [67]. Both the increase in number of altered genes in SC fish and the change in direction of the PCB effect with time likely reflect changes that are secondary to toxicity, as noted above.

To more directly address questions concerning differences in the primary response of SC and NBH fish to PCB, it is best to consider only the 5 and 10 dpf sampling times, prior to the onset of gross embryotoxicity. In looking at these comparisons, it is useful to imagine the different types of gene expression patterns that might be expected under different mechanisms of resistance. Whitehead *et al. *[68] proposed three broad categories of mechanisms underlying tolerance to dioxin-like compounds in *Fundulus*; these, along with two additional scenarios, are illustrated in Additional file 7: Fig. S3. One possible outcome is a complete overlap in gene expression profiles between the two populations, indicating no difference in responsiveness (Fig. S3a). A second theoretical result, no genes with PCB-altered expression in the resistant NBH fish, would result from an AHR-null phenotype or a phenotype with genome-wide desensitization of AHR signaling (Fig. S3b). A third possibility is that only a subset of genes that are PCB-responsive in the SC fish is altered in the NBH fish (Fig. S3c). This type of pattern would be like that seen in a comparison of TCDD-sensitive and -resistant strains of rats [62]. Other possible patterns include gene sets that are partially overlapping (Fig. S3d) or completely distinct (Fig. S3e).

At both 5 and 10 dpf, the number of significantly altered genes was about 3-fold greater for SC embryos as compared to NBH embryos (9 vs. 3 at 5 dpf; 26 vs. 8 at 10 dpf). Interestingly, however, there was no overlap between SC and NBH populations in the PCB-induced or repressed genes at these time points. Thus, some PCB-induced changes occur in NBH fish, but the set of PCB-altered genes in these embryos is not a subset of the set of PCB-altered genes in SC fish. This pattern resembles that in Figure S3e rather than those we considered most likely--the AHR-null pattern (Fig. S3b) or subset pattern (Fig. S3c). However, it is worth noting that the genes induced or repressed in NBH fish exhibited more modest changes as compared to those in SC fish (Additional file 5: Table S3). In addition, the statistical support for the effects in NBH fish was not as strong as for those in SC fish. Slight changes in the statistical cutoff for significance affected the number of significant genes in NBH more than in SC. For example, if the statistical cutoff for significance is changed from p < 0.00669 to p < 0.005, most of the significant genes disappear from the NBH gene sets, with minimal effect on the SC gene sets. Thus, slight adjustments in the statistical stringency can make the pattern resemble more closely the expected "AHR-null" pattern (Fig. S3b). Moreover, even in the case of AHR-null mice, there is a small set of genes altered by TCDD exposure even in the absence of a functioning AHR [67]. Our results are thus consistent with a genome-wide down-regulation of AHR-dependent signaling.

A similar conclusion was reached recently by Whitehead and colleagues, who reported a "global blockade" of the AHR signaling pathway in a different population of PCB-tolerant killifish using a different microarray technology [68]. Of the set of seven "AHR-mediated" genes that showed differential regulation between the sensitive and tolerant populations studied by those authors, two that were on our array (CYP1A and CYP1B1) and two that were identified from our EST libraries (cytochrome b5 and S100 calcium-binding protein A11) showed a similar pattern of induction by PCB in the sensitive SC population but not in the resistant NBH fish. The other three "AHR-mediated" genes identified by Whitehead *et al*. (UDPGT, JUN, and IGFBP1) did not appear to be induced in SC or NBH fish as measured either on our array or in the EST libraries but they were represented by only a small number of reads in the deep sequencing data.

Although our results and those of others [68] are consistent with a genome-wide down-regulation of AHR-dependent signaling in PCB-tolerant fish, there are some additional points that should be considered. First, NBH killifish are not AHR-null, because increasing the dose of inducer is able to overcome the insensitivity and cause altered gene expression and toxicity [8,14,15]. This pattern of reduced sensitivity is more like that seen for the so-called "non-responsive" strains of mice, which are approximately 15-fold less sensitive to TCDD [69,70]. Second, the set of AHR-regulated genes in *Fundulus *is not known. Strong evidence for AHR regulation, in the form of promoter analysis or characterization of AHR response elements, has been obtained only for *Fundulus *CYP1A [71] and AHRR [66]. Third, *F. heteroclitus *has at least two AHRs [72,73]; whether both are suppressed in PCB-tolerant populations is not clear. Recent evidence points to AHR2 as having the primary role in mediating toxicity and altered gene expression caused by PCB-126 in zebrafish embryos [60] and the embryotoxicity of PCB-126 and benzo[k]fluoranthene in killifish embryos [74]. In addition, population genetic analysis of NBH and SC populations implicates the AHR2 gene as being under selection in NBH [25]. Similarly, an AHR2 variant was recently identified as being responsible for the PCB resistance of a population of another species, Atlantic tomcod (*Microgadus tomcod*), inhabiting a PCB-contaminated site--the Hudson River [75]. Thus, AHR2 currently is the most likely candidate for altered function in NBH fish. Distinctions in the sets of genes altered by PCB in SC and NBH fish could reflect divergent changes in the functions of AHR1 and AHR2 in these populations.

## Conclusions

The data presented here provide strong support for the idea that the resistance to PCB-altered gene expression in NBH extends beyond CYP1A and involves many other genes. The results suggest that NBH fish possess a gene regulatory defect that is not specific to one target gene such as CYP1A but rather lies in a regulatory pathway that controls the transcriptional response of multiple genes to PCB exposure. The AHR-dependent signaling pathway is a prime candidate, but whether the defect involves AHR1, AHR2, or other proteins involved in AHR-dependent signaling is not fully understood. Suppression of AHR signaling does not appear to involve up-regulation of the AHR repressor AHRR [66,76] or epigenetic changes in the regulation of AHR expression [20,24,76]. However, both AHR2 and (to a lesser extent) AHR1 show evidence of non-neutral evolution suggestive of selection in the NBH population [25,77], consistent with the possibility that the presence of AHR variants with altered function is responsible for the PCB resistance of NBH fish. AHR target genes that are differentially regulated in these two populations (especially those induced by PCB in SC but not NBH fish) could have a role in toxicity, but whether any of the PCB-regulated genes identified in this study are directly involved in mediating PCB-dependent embryotoxicity in SC fish remains to be determined. Recent advances in adapting loss-of-function approaches to *Fundulus *[30,74] provide an avenue to testing hypotheses about the physiological and toxicological roles of AHR1, AHR2, and specific AHR-regulated genes identified in this study or by others [68].

## Authors' contributions

MFO helped design the study, performed the microarray assays and analyses, helped to draft the manuscript, and helped revise the manuscript. SIK prepared the shotgun deep sequencing library, analyzed the deep sequencing data, and helped revise the manuscript. MJJ helped design the study, performed the dosing experiment and real-time quantitative RT-PCR assays and helped revise the manuscript. DGF assisted with the annotation of genes on the array. DBMW analyzed the deep sequencing data and helped revise the manuscript. MEH helped design the study, assisted with data analysis, helped to draft the manuscript, and helped revise the manuscript. All authors read and approved the final manuscript.

## Supplementary Material

Additional file 1 **Figure S1. Loop design for microarray hybridizations. See text for details**. Click here for file

Additional file 2 **Table S1. Significant differently expressed genes: Results from 2-way ANOVA**. Genes significantly differently expressed at 5, 10 and 15 days post-fertilization. Gene, function, relative fold-differences and p-values are reported. A gene with a positive fold-difference is more highly expressed in population/treatment listed first, and a gene with a negative fold-difference is more highly expressed in the population/treatment listed last. Significant p-values are in **bold**. NBH: New Bedford Harbor (PCB-contaminated site); SC: Scorton Creek (reference site). Unannotated genes are denoted by UnAn and a unique number. Some of the unannotated probes were subsequently annotated after extension using the 454 databases (EST and shotgun libraries); see Table S4 (Additional file 6) for details. Click here for file

Additional file 3 **Figure S2. Volcano plots illustrating gene expression differences at 5, 10, and 15 dpf**. Significances of differences are plotted as -log_10_(p-values) against log_2 _differences in expression. Gene expression differences between **A**. NBH PCB and NBH DMSO treated embryos, **B**. NBH DMSO and SC DMSO treated embryos, **C**. SC PCB and NBH DMSO treated embryos, **D**. NBH PCB and SC DMSO treated embryos, **E**. SC PCB and NBH PCB treated embryos, and **F**. SC PCB and SC DMSO treated embryos. Dashed line demarks the FDR p-value of < 0.01 (p < 0.00669). Click here for file

Additional file 4 **Table S2. Differential gene expression and PCB inducibility in pairwise comparisons of NBH and SC embryos at 5, 10, and 15 dpf**. Genes with significant differences in pairwise comparisons of gene expression are included. Gene expression ratios are indicated. A gene with a positive fold-difference is more highly expressed in the population/treatment listed first, and a gene with a negative fold-difference is more highly expressed in the population/treatment listed last. Genes are listed in order of ratios in the reference population (SC) comparison with tolerant population (SC). Ratios with significant p-values are in **bold**. See Table S1 (Additional file 2) for a list of all genes significant in the ANOVA analysis. NBH: New Bedford Harbor; SC: Scorton Creek; PCB: PCB-126; DMSO: dimethylsulfoxide. Unannotated genes are denoted by UnAn and a unique number. Some of the unannotated probes were subsequently annotated after extension using the 454 database; see Table S4 (Additional file 6) for details. Click here for file

Additional file 5 **Table S3. Mean fold change in gene expression in pairwise comparisons**. Click here for file

Additional file 6 **Table S4. Probes annotated as a result of 454 sequencing**. Microarray probe sequences were used in blast searches against the 454 sequence data (EST and shotgun libraries). The probe sequences that were extended with the matching reads were used to search GenBank using blast to obtain the annotations. Click here for file

Additional file 7 **Figure S3**. Possible scenarios comparing the response of SC and NBH fish to PCB. Click here for file
