# Supplementary figures and images for: Transcriptomic assessment of resistance to effects of an aryl hydrocarbon receptor (AHR) agonist in embryos of Atlantic killifish (Fundulus heteroclitus) from a marine Superfund site
Source: BMC Genomics. 2011 May 24;12:263. doi: 10.1186/1471-2164-12-263 (PMC3213123; doi:10.1186/1471-2164-12-263)

Figure S1

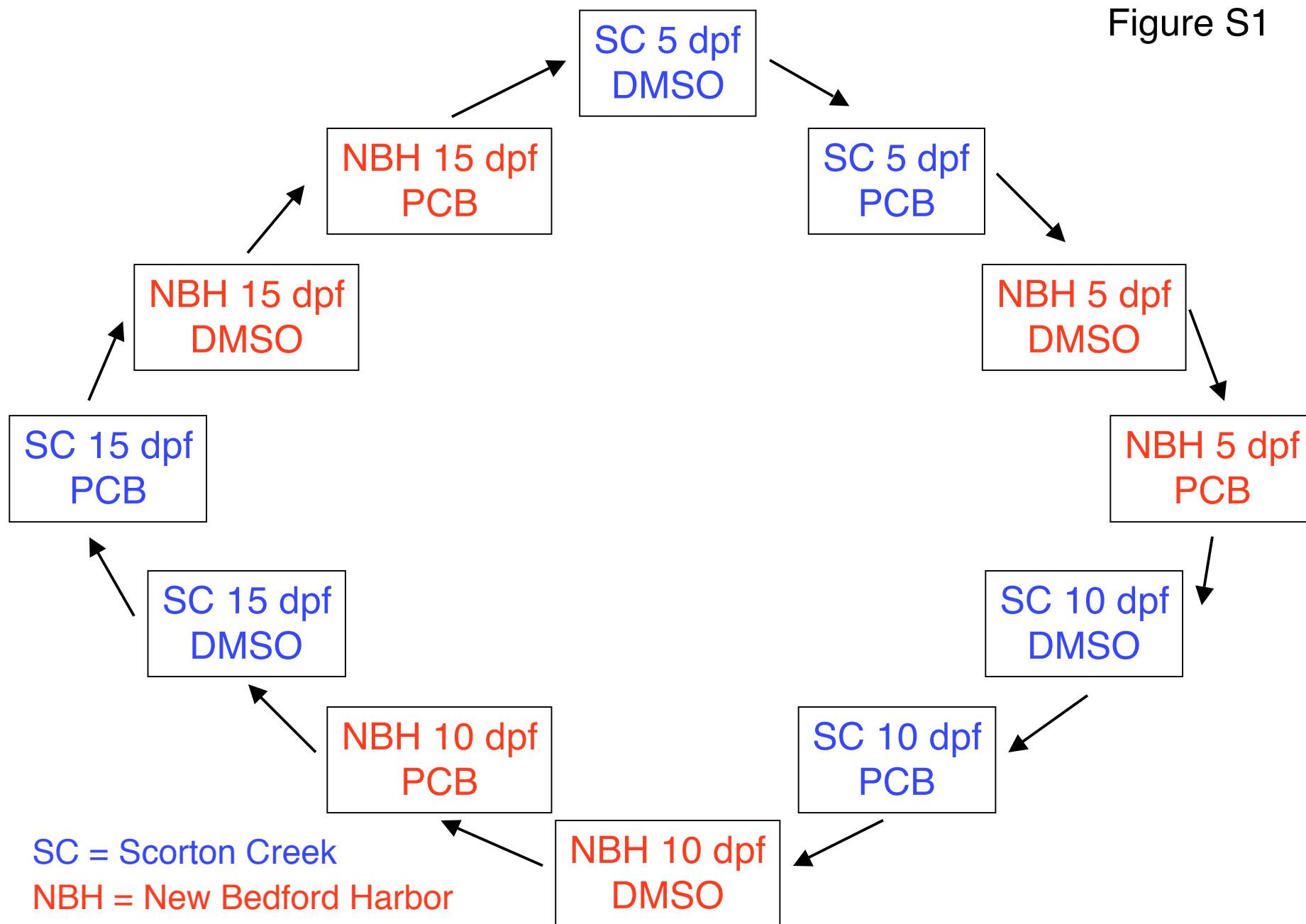

Supplement: Additional file 1 — Figure S1. Loop design for microarray hybridizations. See text for details. [file 1471-2164-12-263-S1.PDF]

Figure S2

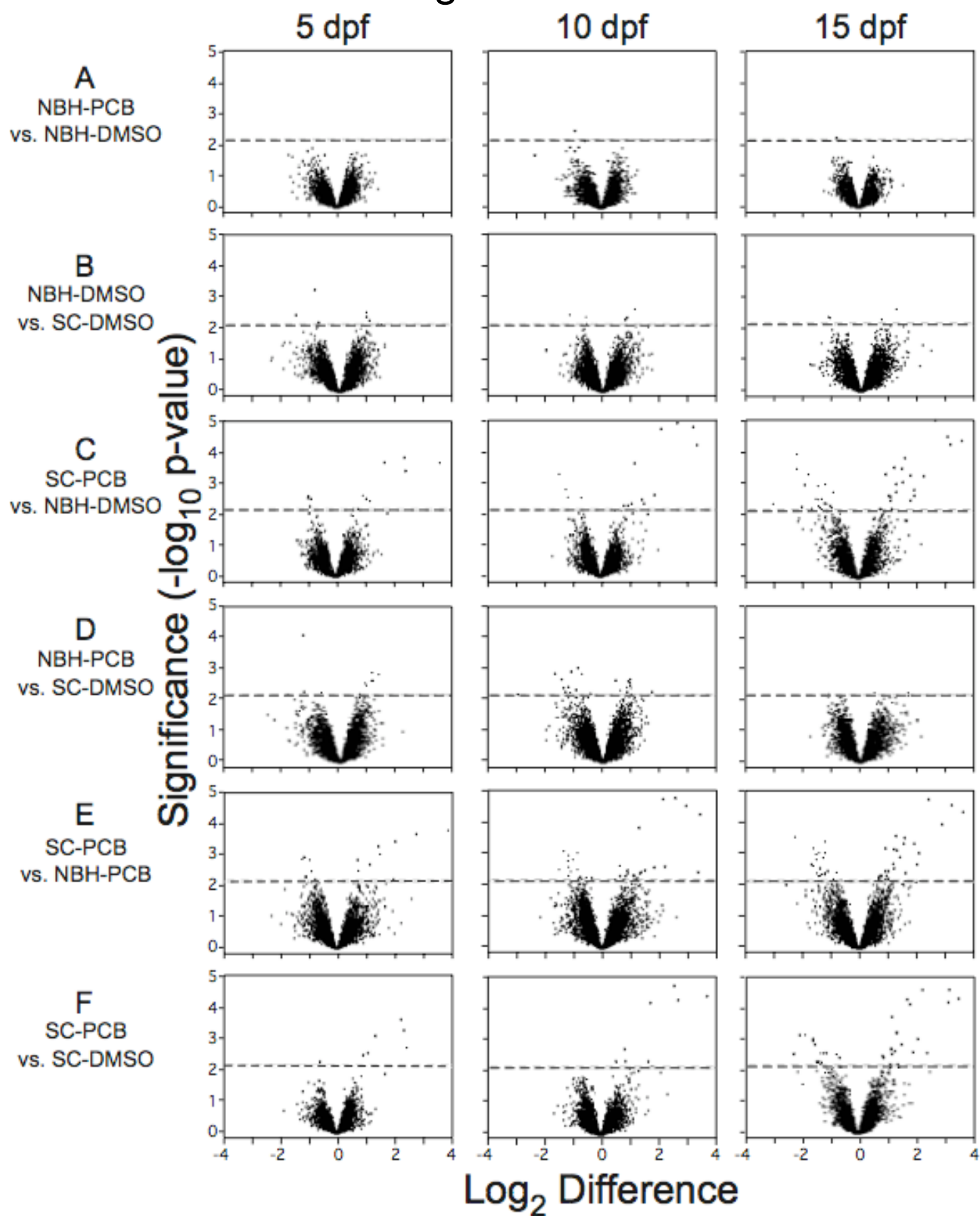

Supplement: Additional file 3 — Figure S2. Volcano plots illustrating gene expression differences at 5, 10, and 15 dpf. Significances of differences are plotted as -log10(p-values) against log2 differences in expression. Gene expression differences between A. NBH PCB and NBH DMSO treated embryos, B. NBH DMSO and SC DMSO treated embryos, C. SC PCB and NBH DMSO treated embryos, D. NBH PCB and SC DMSO treated embryos, E. SC PCB and NBH PCB treated embryos, and F. SC PCB and SC DMSO treated embryos. Dashed line demarks the FDR p-value of < 0.01 (p < 0.00669). [file 1471-2164-12-263-S3.PDF]
