# Supplementary material for: Transcriptomic assessment of resistance to effects of an aryl hydrocarbon receptor (AHR) agonist in embryos of Atlantic killifish (Fundulus heteroclitus) from a marine Superfund site
Source: BMC Genomics. 2011 May 24;12:263. doi: 10.1186/1471-2164-12-263 (PMC3213123; doi:10.1186/1471-2164-12-263)
Supplement: Additional file 2 — Table S1. Significant differently expressed genes: Results from 2-way ANOVA. Genes significantly differently expressed at 5, 10 and 15 days post-fertilization. Gene, function, relative fold-differences and p-values are reported. A gene with a positive fold-difference is more highly expressed in population/treatment listed first, and a gene with a negative fold-difference is more highly expressed in the population/treatment listed last. Significant p-values are in bold. NBH: New Bedford Harbor (PCB-contaminated site); SC: Scorton Creek (reference site). Unannotated genes are denoted by UnAn and a unique number. Some of the unannotated probes were subsequently annotated after extension using the 454 databases (EST and shotgun libraries); see Table S4 (Additional file 6) for details. [file 1471-2164-12-263-S2.DOCX]

Oleksiak et al. BMC Genomics

**Additional file 2: Table S1. Significant differently expressed genes: Results from 2-way ANOVA.**

Genes significantly differently expressed at 5, 10 and 15 days post-fertilization. Gene, function, relative fold-differences and p-values are reported. A gene with a positive fold-difference is more highly expressed in population/treatment listed first, and a gene with a negative fold-difference is more highly expressed in the population/treatment listed last. Significant p-values are in **bold**. NBH are New Bedford Harbor embryos and SC are Scorton Creek (reference) embryos. Unannotated genes are denoted by UnAn and a unique number. Some of the unannotated probes were subsequently annotated after extension using the 454 database; see Table S3 for details.

| **Gene** | **NBH PCB vs. NBH DMSO** | **NBH DMSO vs. SC DMSO** | **SC PCB vs. NBH DMSO** | **NBH PCB vs. SC DMSO** | **SC PCB vs. NBH PCB** | **SC PCB vs. SC DMSO** | **P-value Population** | **P-value Treatment** | **P-value Population by Treatment** |
| --- | --- | --- | --- | --- | --- | --- | --- | --- | --- |
| **5 Days Post-fertilization** |  |  |  |  |  |  |  |  |  |
| ATP synthase lipid-binding protein | 1.79 | 1.47 | -1.12 | 2.64 | -2.02 | 1.31 | 1.09E-02 | **4.60E-03** | 1.54E-01 |
| basic leucine zipper nuclear factor 1 | -1.03 | -1.52 | 7.74 | -1.57 | 7.97 | 5.09 | **3.00E-04** | **3.00E-04** | **3.00E-04** |
| Complement component C9 precursor | 1.48 | 1.69 | -1.40 | 2.50 | -2.08 | 1.21 | **2.00E-03** | 3.59E-02 | 3.97E-01 |
| Complex I-B9 | 1.23 | 1.49 | -1.41 | 1.84 | -1.73 | 1.06 | **1.60E-03** | 1.04E-01 | 3.72E-01 |
| CYP1A | 1.29 | -1.12 | 5.40 | 1.15 | 4.18 | 4.82 | **1.60E-03** | **6.00E-04** | **3.40E-03** |
| CYP1B1 | -1.21 | -1.26 | 2.06 | -1.53 | 2.5 | 1.64 | **3.20E-03** | 1.47E-01 | 1.24E-02 |
| Deoxyribonuclease-1 | 1.48 | -2.57 | 2.25 | -1.74 | 1.52 | -1.14 | **5.40E-03** | 2.79E-01 | 5.51E-02 |
| Diablo homolog | -1.03 | 2.14 | 1.12 | 2.07 | 1.16 | 2.40 | 3.78E-01 | 5.01E-02 | **5.10E-03** |
| Guanidinoacetate N-methyltransferase | -1.16 | -3.28 | 2.62 | -3.82 | 3.05 | -1.25 | **1.40E-03** | 1.77E-01 | 7.51E-01 |
| hect domain and RLD 4 isoform b | 1.33 | -1.06 | 1.28 | 1.25 | -1.04 | 1.21 | 8.95E-01 | **4.60E-03** | 2.66E-01 |
| Hypothetical protein C31G5.21 | 1.12 | 1.98 | -1.57 | 2.22 | -1.75 | 1.27 | **1.10E-03** | 3.93E-02 | 3.54E-01 |
| IGF-II mRNA-binding protein 2 isoform a | -1.85 | -1.44 | 1.28 | -2.65 | 2.37 | -1.12 | **6.40E-03** | 1.77E-02 | 8.22E-02 |
| Prostaglandin G/H synthase 1 precursor | 1.02 | 1.43 | -1.49 | 1.46 | -1.52 | -1.04 | **3.90E-03** | 8.68E-01 | 5.52E-01 |
| Proteasome subunit beta type 7 | 1.41 | -1.14 | 1.03 | 1.24 | -1.37 | -1.10 | 4.14E-01 | 9.04E-02 | **4.60E-03** |
| Small inducible cytokine A4 homolog | 1.13 | 1.03 | 1.72 | 1.17 | 1.52 | 1.78 | 7.69E-02 | **9.00E-04** | **6.50E-03** |
| Synaptophysin-like protein | -1.15 | 1.64 | -1.14 | 1.42 | 1.01 | 1.43 | 1.22E-01 | 1.81E-01 | **1.70E-03** |
| T-cell surface glycoprotein CD3 delta chain | 1.14 | -1.64 | 4.80 | -1.44 | 4.21 | 2.92 | **4.50E-03** | **6.50E-03** | 2.07E-02 |
| T-cell surface glycoprotein CD8 beta chain | 1.06 | 1.59 | -1.63 | 1.68 | -1.72 | -1.03 | **4.20E-03** | 8.57E-01 | 6.61E-01 |
| U2-associated SR140 protein | 1.21 | 1.32 | -1.46 | 1.59 | -1.77 | -1.11 | **1.90E-03** | 4.92E-01 | 5.05E-02 |
| UnAn_21996 | 1.28 | 1.02 | 1.81 | 1.30 | 1.42 | 1.84 | 1.84E-01 | **6.00E-04** | 1.45E-02 |
| UnAn_22354 | -1.05 | 2.30 | -2.29 | 2.20 | -2.19 | 1.00 | **2.80E-03** | 8.77E-01 | 8.63E-01 |
| UnAn_22456 | -1.08 | 1.60 | -1.67 | 1.49 | -1.55 | -1.04 | **6.20E-03** | 4.52E-01 | 8.23E-01 |
| UnAn_22681 | 1.38 | 1.47 | -1.41 | 2.02 | -1.94 | 1.04 | **2.40E-03** | 4.55E-02 | 1.07E-01 |
| UnAn_22726 | 1.03 | 1.88 | -2.07 | 1.93 | -2.12 | -1.10 | **3.00E-04** | 5.72E-01 | 3.37E-01 |
| UnAn_23042 | -1.13 | -1.41 | 1.51 | -1.60 | 1.71 | 1.07 | **1.40E-03** | 7.09E-01 | 2.73E-01 |
| UnAn_23246 | -1.09 | 1.96 | -1.90 | 1.81 | -1.75 | 1.03 | **8.00E-04** | 7.33E-01 | 4.98E-01 |
| UnAn_26723 | -1.14 | 2.12 | -1.86 | 1.86 | -1.63 | 1.14 | **5.40E-03** | 9.81E-01 | 1.55E-01 |
| UnAn_27108 | -1.10 | 1.93 | -1.79 | 1.76 | -1.63 | 1.08 | **6.00E-03** | 9.30E-01 | 4.64E-01 |
| UnAn_27722 | 1.18 | 1.52 | -1.53 | 1.80 | -1.82 | -1.01 | **6.00E-03** | 3.30E-01 | 2.64E-01 |
| UnAn_27910 | 1.22 | -1.32 | 3.35 | -1.08 | 2.74 | 2.54 | **7.00E-04** | **3.00E-04** | **3.50E-03** |
| UnAn_29411 | -1.03 | -2.51 | 11.58 | -2.58 | 11.87 | 4.61 | **3.00E-04** | **1.50E-03** | **8.00E-04** |
| UnAn_29726 | 1.01 | -1.23 | 1.54 | -1.22 | 1.53 | 1.25 | **6.20E-03** | 1.41E-01 | 1.84E-01 |
| UnAn_29829 | -1.16 | -1.16 | 1.43 | -1.34 | 1.65 | 1.23 | **6.00E-03** | 5.30E-01 | **5.10E-03** |
| **10 Days Post-fertilization** |  |  |  |  |  |  |  |  |  |
| 60S acidic ribosomal protein P1 | 1.44 | 1.26 | -1.16 | 1.81 | -1.66 | 1.09 | **5.30E-03** | 2.17E-02 | 1.27E-01 |
| Acidic phosphoprotein | 1.11 | -1.15 | 2.73 | -1.04 | 2.46 | 2.37 | 9.00E-03 | **1.40E-03** | **3.70E-03** |
| AMP deaminase 1 | 1.27 | 1.49 | -1.04 | 1.88 | -1.32 | 1.43 | 1.10E-02 | **1.70E-03** | 2.69E-01 |
| Apomucin | 1.16 | 1.45 | 1.39 | 1.68 | 1.2 | 2.02 | 5.51E-01 | **3.50E-03** | 1.53E-02 |
| Beta enolase | -1.00 | -1.29 | -1.19 | -1.29 | -1.19 | -1.53 | 8.16E-01 | 5.11E-02 | **2.70E-03** |
| bromodomain containing 9 isoform 1 | -1.49 | -1.23 | 1.03 | -1.84 | 1.54 | -1.19 | **3.90E-03** | **6.50E-03** | 1.65E-01 |
| C61 protein | 1.22 | 1.84 | 1.08 | 2.25 | -1.13 | 1.99 | 9.64E-02 | **5.50E-03** | 2.26E-02 |
| Cathepsin D | -1.45 | 1.01 | 1.03 | -1.44 | 1.49 | 1.04 | 2.08E-01 | 6.61E-02 | **4.40E-03** |
| Chromobox protein homolog 2 | -1.65 | -1.36 | -1.08 | -2.25 | 1.53 | -1.47 | 6.51E-02 | **3.40E-03** | 4.83E-01 |
| CIITA | 1.03 | -1.06 | 1.86 | -1.03 | 1.82 | 1.76 | 1.31E-02 | **5.80E-03** | 1.20E-02 |
| CYP1A | 1.10 | -1.13 | 6.91 | -1.03 | 6.26 | 6.10 | **1.00E-04** | **4.10E-05** | **1.00E-04** |
| Cytochrome b | -1.72 | -1.53 | -1.03 | -2.63 | 1.67 | -1.57 | 1.86E-02 | **2.60E-03** | 6.91E-01 |
| EGLP | -1.73 | -1.32 | 1.15 | -2.29 | 1.98 | -1.15 | **1.20E-03** | **6.00E-04** | **6.00E-03** |
| Epsin-4 | 1.38 | 2.03 | -1.25 | 2.80 | -1.73 | 1.62 | 8.40E-03 | **4.10E-03** | 2.53E-01 |
| Histidine-rich glycoprotein | -1.16 | -1.52 | 1.50 | -1.77 | 1.75 | -1.01 | **3.10E-03** | 2.90E-01 | 3.81E-01 |
| MEK binding partner 1 | -1.10 | -1.51 | 1.41 | -1.66 | 1.55 | -1.07 | **5.00E-04** | 2.42E-01 | 8.44E-01 |
| Muted protein | -1.60 | -1.50 | 1.29 | -2.41 | 2.08 | -1.16 | **4.50E-03** | 7.90E-03 | 7.33E-02 |
| NADH dehydrogenase subunit 5 | -1.75 | -1.82 | 1.48 | -3.18 | 2.59 | -1.23 | **1.90E-03** | 3.99E-02 | 2.70E-01 |
| Parvalbumin beta | 1.30 | -1.49 | -1.21 | -1.14 | -1.57 | -1.80 | 9.06E-01 | 2.23E-01 | **7.00E-04** |
| quiescin Q6 isoform a | -1.44 | -1.34 | 2.24 | -1.94 | 3.24 | 1.67 | 2.64E-02 | 6.07E-01 | **2.40E-03** |
| RWD domain containing protein 1 | 1.05 | -1.05 | -1.65 | -1.00 | -1.73 | -1.73 | 1.80E-01 | 3.08E-02 | **2.80E-03** |
| snRNP core protein D2 | -1.21 | -1.12 | -1.08 | -1.35 | 1.12 | -1.21 | 7.49E-02 | **3.70E-03** | 9.43E-01 |
| T-cell receptor T3 delta chain | 1.07 | -1.25 | 6.81 | -1.16 | 6.35 | 5.47 | **1.20E-03** | **4.00E-04** | **7.00E-04** |
| T-cell surface glycoprotein CD8 beta chain | 1.51 | -1.12 | -1.18 | 1.35 | -1.78 | -1.32 | 1.04E-01 | 3.80E-01 | **2.00E-03** |
| Transcription factor PU.1 | 1.19 | -1.36 | -1.13 | -1.14 | -1.35 | -1.55 | 9.67E-01 | 1.19E-01 | **5.80E-03** |
| Troponin C | 1.08 | 2.68 | -1.64 | 2.90 | -1.77 | 1.63 | **6.60E-03** | 5.47E-02 | 1.22E-01 |
| Tuberin | -1.55 | -1.75 | 1.3 | -2.72 | 2.02 | -1.35 | **3.70E-03** | **5.80E-03** | 4.39E-01 |
| Tubulointerstitial nephritis antigen-like precursor | 1.49 | -1.03 | 3.09 | 1.44 | 2.08 | 3.00 | 1.79E-01 | **1.40E-03** | **3.80E-03** |
| Type II antifreeze protein | -1.12 | 1.72 | -2.25 | 1.54 | -2.00 | -1.30 | **8.00E-04** | 3.70E-02 | 3.63E-01 |
| Tyrosine aminotransferase | -1.14 | -1.24 | 2.35 | -1.40 | 2.68 | 1.90 | **2.00E-04** | **1.70E-03** | **1.00E-04** |
| Ubiquinol-cytochrome c reductase iron-sulfur subunit | -1.03 | -1.74 | 1.70 | -1.78 | 1.74 | -1.02 | **5.40E-03** | 7.38E-01 | 9.74E-01 |
| UnAn_20648 | 1.32 | 1.01 | -1.06 | 1.32 | -1.39 | -1.05 | 1.26E-01 | 7.67E-02 | **5.40E-03** |
| UnAn_20957 | -1.84 | -1.48 | 1.12 | -2.71 | 2.06 | -1.32 | 1.85E-02 | **4.10E-03** | 7.01E-02 |
| UnAn_21518 | -1.00 | -1.57 | 1.57 | -1.58 | 1.57 | -1.00 | **3.60E-03** | 9.68E-01 | 1.00E+00 |
| UnAn_21996 | -1.15 | -1.01 | 2.10 | -1.16 | 2.42 | 2.09 | 1.75E-02 | 1.48E-02 | **2.30E-03** |
| UnAn_22354 | -1.09 | 2.13 | -2.72 | 1.95 | -2.49 | -1.28 | **2.00E-04** | 1.59E-01 | 4.83E-01 |
| UnAn_22447f | 1.34 | 1.27 | -1.30 | 1.70 | -1.75 | -1.03 | **3.20E-03** | 6.04E-02 | 4.09E-02 |
| UnAn_22666 | 1.13 | 1.61 | -1.65 | 1.83 | -1.87 | -1.03 | **5.80E-03** | 5.82E-01 | 4.03E-01 |
| UnAn_22785 | -1.24 | -1.84 | 1.82 | -2.27 | 2.25 | -1.01 | **7.00E-04** | 1.24E-01 | 6.55E-02 |
| UnAn_22873 | -1.00 | 1.30 | -2.01 | 1.29 | -2.00 | -1.55 | **4.40E-03** | 2.21E-02 | 2.61E-02 |
| UnAn_22879 | 1.56 | -1.51 | 1.25 | 1.03 | -1.25 | -1.20 | 4.63E-01 | 1.51E-01 | **6.20E-03** |
| UnAn_23038 | 1.17 | 1.15 | -1.08 | 1.35 | -1.27 | 1.06 | **3.60E-03** | 3.59E-02 | 2.88E-01 |
| UnAn_23120 | 1.37 | 1.35 | -1.55 | 1.85 | -2.12 | -1.14 | **1.40E-03** | 2.54E-01 | 2.58E-02 |
| UnAn_23121 | -1.09 | 1.54 | 1.14 | 1.41 | 1.24 | 1.75 | 5.27E-01 | 4.41E-02 | **6.00E-03** |
| UnAn_23610r | 1.06 | -1.45 | 3.34 | -1.37 | 3.15 | 2.30 | 1.10E-02 | 8.20E-03 | **2.80E-03** |
| UnAn_27466 | 1.30 | -1.33 | -1.16 | -1.03 | -1.50 | -1.54 | 5.51E-01 | 2.30E-01 | **1.80E-03** |
| UnAn_27910 | -1.02 | -1.29 | 4.34 | -1.32 | 4.44 | 3.36 | **1.00E-04** | **2.00E-04** | **3.00E-04** |
| UnAn_27985 | -1.10 | 1.13 | 1.27 | 1.03 | 1.40 | 1.44 | 1.18E-01 | 4.14E-02 | **5.50E-03** |
| UnAn_28041 | 1.02 | -1.01 | 2.45 | 1.01 | 2.42 | 2.43 | 8.90E-03 | **6.00E-04** | **2.00E-04** |
| UnAn_28436 | 1.13 | 1.29 | -1.46 | 1.46 | -1.66 | -1.14 | **1.30E-03** | 9.73E-01 | 6.15E-02 |
| UnAn_28540 | -1.17 | 1.04 | -1.37 | -1.12 | -1.17 | -1.31 | 2.52E-01 | **5.20E-03** | 2.80E-01 |
| UnAn_29159 | 1.18 | -1.20 | 3.20 | -1.02 | 2.71 | 2.66 | 1.36E-02 | **3.40E-03** | 1.97E-02 |
| UnAn_29343 | 1.20 | 1.48 | -1.13 | 1.77 | -1.35 | 1.31 | 7.80E-03 | **4.70E-03** | 3.22E-01 |
| UnAn_29411 | 1.09 | 1.12 | 10.55 | 1.22 | 9.69 | 11.78 | **8.00E-04** | **1.00E-04** | **1.00E-04** |
| UnAn_29819 | 1.37 | 1.28 | -1.34 | 1.76 | -1.84 | -1.05 | **3.50E-03** | 7.42E-02 | 3.15E-02 |
| UnAn_29849 | 1.11 | 1.67 | -1.35 | 1.86 | -1.50 | 1.24 | **1.80E-03** | 3.64E-02 | 4.36E-01 |
| WAP four-disulfide core domain protein 3 | 1.02 | 1.59 | 1.23 | 1.63 | 1.21 | 1.96 | 6.26E-01 | 4.95E-02 | **5.50E-03** |
| **15 Days Post-fertilization** |  |  |  |  |  |  |  |  |  |
| 3-ketoacyl-CoA thiolase | -1.19 | -1.30 | 1.47 | -1.54 | 1.75 | 1.13 | 9.40E-03 | 6.93E-01 | **6.00E-04** |
| 30S ribosomal protein S12 | 1.02 | 1.14 | -1.65 | 1.16 | -1.68 | -1.45 | 4.30E-02 | 3.53E-02 | **4.00E-04** |
| 40S ribosomal protein S6 | -1.13 | -1.24 | 1.49 | -1.40 | 1.68 | 1.20 | **6.20E-03** | 6.73E-01 | 9.38E-02 |
| Acidic phosphoprotein | 1.13 | -1.11 | -1.72 | 1.03 | -1.95 | -1.90 | 5.01E-02 | 7.90E-03 | **3.00E-04** |
| Acidic phosphoprotein | -1.09 | -1.09 | 4.79 | -1.19 | 5.22 | 4.40 | **2.40E-03** | **4.00E-04** | **4.70E-05** |
| ADP/ATP translocase 3 | -1.03 | -1.02 | -1.75 | -1.05 | -1.70 | -1.78 | 4.97E-02 | **6.20E-03** | 1.25E-02 |
| Aminoacylase-1 | -1.19 | -1.28 | -1.22 | -1.52 | -1.02 | -1.55 | 3.04E-01 | **5.30E-03** | 1.38E-01 |
| Angiogenic factor VG5Q | -1.04 | 1.16 | 1.54 | 1.12 | 1.60 | 1.80 | 1.66E-01 | **5.10E-03** | **2.10E-03** |
| Apomucin | 1.07 | -1.70 | -1.74 | -1.59 | -1.86 | -2.96 | 7.33E-01 | **1.80E-03** | **1.40E-03** |
| Atrial natriuteric peptide-converting enzyme | 1.13 | -1.24 | 3.49 | -1.09 | 3.08 | 2.82 | 7.60E-03 | **1.10E-03** | **1.90E-03** |
| basic leucine zipper nuclear factor 1 | 1.30 | 1.17 | 3.08 | 1.52 | 2.36 | 3.60 | 1.00E-02 | **1.00E-04** | **1.40E-03** |
| Brain mitochondrial carrier protein-1 | -1.11 | 1.10 | -1.84 | -1.01 | -1.65 | -1.67 | 5.00E-02 | **5.80E-03** | 2.15E-02 |
| BTG1 protein | 1.04 | -1.27 | 2.16 | -1.22 | 2.08 | 1.70 | 1.12E-02 | **6.00E-03** | **3.90E-05** |
| C61 protein | -1.01 | 1.12 | 1.49 | 1.11 | 1.51 | 1.67 | 3.17E-01 | 1.78E-02 | **5.00E-03** |
| Catalase | 1.34 | -1.00 | 1.91 | 1.34 | 1.42 | 1.91 | 3.99E-01 | **4.10E-03** | 3.14E-02 |
| Catalase | 1.13 | -1.11 | 2.44 | 1.02 | 2.15 | 2.20 | 1.56E-02 | **2.70E-03** | 1.51E-02 |
| Cathepsin Z | 1.12 | 1.04 | -2.08 | 1.17 | -2.33 | -2.00 | 4.64E-02 | 3.47E-02 | **4.80E-03** |
| CIITA | 1.16 | -1.07 | 1.71 | 1.08 | 1.48 | 1.60 | 6.71E-02 | **2.80E-03** | 3.05E-02 |
| Collagen alpha 1(X) chain | -1.12 | 1.31 | -3.38 | 1.16 | -3.01 | -2.59 | **1.80E-03** | **3.00E-04** | **2.00E-04** |
| Collagen alpha 2(I) chain | 1.24 | 1.14 | -2.24 | 1.41 | -2.77 | -1.96 | 9.10E-03 | 2.93E-02 | **1.00E-04** |
| Complement C1q tumor necrosis factor-related protein 5 | -1.10 | 1.74 | -1.99 | 1.58 | -1.81 | -1.15 | **2.50E-03** | 2.61E-01 | 8.47E-01 |
| Complement C4 | -1.07 | -1.74 | 1.92 | -1.86 | 2.05 | 1.10 | **5.70E-03** | 8.89E-01 | 4.05E-01 |
| complement component 3 | 1.65 | 1.86 | -1.16 | 3.08 | -1.93 | 1.60 | 1.12E-02 | **3.70E-03** | 8.57E-01 |
| Complement component C8 beta chain | -1.37 | -1.14 | -1.37 | -1.56 | 1.00 | -1.56 | 5.47E-01 | **6.00E-04** | 2.14E-01 |
| Complement factor H-related protein 1 | 1.05 | 1.05 | 1.53 | 1.10 | 1.45 | 1.60 | 1.73E-01 | **5.50E-03** | 6.70E-03 |
| CXXC finger 5 | -1.05 | -1.11 | 1.79 | -1.16 | 1.88 | 1.62 | 1.90E-02 | 1.43E-02 | **8.00E-04** |
| CYP1A | -1.19 | 1.17 | 8.78 | -1.02 | 10.46 | 10.27 | **2.00E-04** | **2.10E-05** | **1.10E-05** |
| CYP2J5 | -1.31 | -1.09 | 1.08 | -1.43 | 1.41 | -1.01 | 8.03E-02 | 4.22E-02 | **4.30E-03** |
| Death-associated protein 1 | 1.15 | -1.02 | 1.59 | 1.12 | 1.39 | 1.56 | 1.93E-01 | **5.50E-03** | 3.11E-02 |
| Desmin | 1.11 | 1.15 | 1.64 | 1.28 | 1.47 | 1.88 | 4.63E-01 | **6.30E-03** | 1.11E-02 |
| DNA topoisomerase I | 1.03 | 1.80 | -2.11 | 1.84 | -2.17 | -1.18 | **3.70E-03** | 5.46E-01 | 4.35E-01 |
| DNase II alpha | 1.08 | 1.06 | 1.17 | 1.15 | 1.08 | 1.25 | 9.10E-01 | 1.86E-02 | **3.00E-04** |
| Dual specificity protein kinase CLK1 | -1.05 | 1.12 | 1.30 | 1.07 | 1.36 | 1.45 | 4.90E-01 | 6.80E-02 | **5.00E-03** |
| Ependymin | -1.07 | 1.13 | 1.99 | 1.06 | 2.13 | 2.25 | 1.65E-02 | **7.00E-04** | **2.00E-04** |
| ETHE1 protein | -1.29 | -1.03 | 2.22 | -1.32 | 2.87 | 2.17 | **2.80E-03** | 3.41E-02 | **2.20E-03** |
| Ferritin M | -1.30 | 1.13 | 1.46 | -1.15 | 1.89 | 1.64 | 9.61E-02 | 1.85E-01 | **2.10E-03** |
| Glutathione S-transferase 1 | -1.42 | -1.84 | 2.24 | -2.62 | 3.18 | 1.21 | 1.34E-02 | 5.78E-01 | **6.40E-03** |
| Glycogen synthase kinase-3 alpha | -1.32 | -1.28 | -1.10 | -1.69 | 1.20 | -1.41 | 5.01E-02 | **2.60E-03** | 6.36E-01 |
| Guanine nucleotide-binding protein G(T) gamma-T1 subunit | 1.19 | 1.10 | -1.85 | 1.31 | -2.20 | -1.68 | **9.00E-04** | 1.31E-02 | **5.00E-04** |
| Homogentisate 1,2-dioxygenase | 1.12 | 1.05 | 1.60 | 1.18 | 1.42 | 1.69 | 1.17E-01 | **3.00E-04** | **1.00E-04** |
| Hypothetical 23.7 kDa protein in MDH1-VMA5 intergenic region | -1.31 | -1.15 | 1.90 | -1.51 | 2.48 | 1.65 | 6.56E-02 | 3.78E-01 | **8.00E-04** |
| Hypothetical 26.3 kDa protein in RAD4-CHD1 intergenic region | 1.14 | -1.11 | 3.02 | 1.03 | 2.65 | 2.72 | **4.70E-03** | **8.00E-04** | **4.20E-03** |
| hypothetical protein LOC196463 | -1.05 | -1.10 | -1.42 | -1.15 | -1.35 | -1.56 | 5.22E-01 | 2.35E-02 | **5.60E-03** |
| hypothetical protein LOC76747 | -1.07 | 1.03 | 1.77 | -1.03 | 1.89 | 1.83 | 3.23E-02 | **6.50E-03** | **1.30E-03** |
| Isocitrate dehydrogenase | -1.18 | 1.69 | 1.13 | 1.43 | 1.33 | 1.91 | 6.50E-01 | 1.19E-01 | **2.30E-03** |
| Keratin, type I | -1.4 | -1.21 | -1.42 | -1.69 | -1.01 | -1.71 | 4.82E-01 | **8.00E-04** | 1.62E-01 |
| LDH-B | 1.22 | -1.08 | 1.73 | 1.13 | 1.43 | 1.61 | 9.81E-02 | **2.00E-03** | 4.63E-02 |
| Lymphocyte antigen Ly-6D | -1.19 | 1.49 | 1.35 | 1.25 | 1.61 | 2.01 | 7.86E-01 | 3.16E-02 | **4.40E-03** |
| lymphocyte specific 1 | 1.07 | 1.55 | -1.42 | 1.66 | -1.52 | 1.09 | **3.00E-03** | 2.27E-01 | 8.43E-01 |
| Magnesium-chelatase subunit chlI | 1.06 | -1.06 | 1.42 | -1.00 | 1.34 | 1.34 | 1.49E-01 | 1.98E-02 | **1.70E-03** |
| Major vault protein | -1.24 | 1.16 | 1.18 | -1.07 | 1.46 | 1.36 | 2.08E-01 | 4.19E-01 | **1.90E-03** |
| Microsomal glutathione S-transferase 3 | 1.05 | 1.37 | 1.19 | 1.44 | 1.13 | 1.63 | 3.61E-01 | **5.00E-03** | 1.22E-02 |
| Myosin-binding protein C | 1.26 | -1.18 | -1.71 | 1.07 | -2.16 | -2.02 | 8.66E-02 | 7.02E-02 | **6.50E-03** |
| NDRG1 protein | 1.03 | 1.56 | 1.33 | 1.61 | 1.29 | 2.07 | 4.97E-01 | **4.20E-03** | **6.30E-03** |
| Nuclear protein 1 | 1.3 | -1.15 | 1.45 | 1.13 | 1.12 | 1.26 | 2.81E-01 | **4.00E-03** | 5.93E-01 |
| Parvalbumin beta | -1.02 | -1.10 | -3.56 | -1.12 | -3.48 | -3.9 | 6.42E-02 | **5.00E-03** | **4.80E-03** |
| Parvalbumin beta | -1.22 | 1.04 | -2.80 | -1.18 | -2.30 | -2.71 | 2.91E-02 | **1.00E-03** | 7.30E-03 |
| Parvalbumin beta | 1.08 | 1.19 | -4.44 | 1.29 | -4.82 | -3.72 | **7.00E-04** | **1.20E-03** | **9.00E-04** |
| Periplakin | 1.38 | 1.01 | -1.87 | 1.40 | -2.57 | -1.84 | **4.60E-03** | 2.34E-01 | **5.30E-03** |
| PGE receptor, EP4 subtype | -1.30 | -1.00 | 1.71 | -1.30 | 2.23 | 1.71 | 2.71E-02 | 1.37E-01 | **8.00E-04** |
| Phosphodeoxyriboaldolase | 1.11 | -1.07 | -1.23 | 1.04 | -1.36 | -1.31 | 1.96E-01 | 1.19E-01 | **1.10E-03** |
| Plasma serine protease inhibitor | 1.09 | 1.02 | -1.94 | 1.10 | -2.11 | -1.91 | 2.76E-02 | 1.80E-02 | **6.10E-03** |
| Potassium/sodium hyperpolarization-activated cyclic nucleotide-gated channel 2 | -1.10 | 1.22 | 1.24 | 1.11 | 1.36 | 1.51 | 6.13E-01 | 4.76E-02 | **4.20E-03** |
| Pre-mRNA branch site protein p14 | 1.12 | 1.12 | -2.00 | 1.26 | -2.24 | -1.78 | 1.72E-02 | 2.38E-02 | **4.00E-04** |
| Probable pancreatic secretory proteinase inhibitor | -1.03 | 1.39 | 2.37 | 1.35 | 2.44 | 3.29 | 1.54E-02 | **3.00E-04** | **3.00E-04** |
| Proteasome subunit alpha type 2 | 1.33 | 1.31 | 1.65 | 1.75 | 1.24 | 2.17 | 8.54E-01 | **2.70E-03** | 7.67E-02 |
| Protein C18orf37 homolog | 1.10 | -1.12 | 2.54 | -1.01 | 2.30 | 2.28 | **4.50E-03** | **1.60E-03** | 7.40E-03 |
| Protein C20orf149 | -1.01 | -1.02 | 1.39 | -1.04 | 1.41 | 1.36 | 1.86E-01 | 6.62E-02 | **5.20E-03** |
| Protein disulfide-isomerase A4 | 1.20 | -1.20 | -1.15 | 1.00 | -1.38 | -1.38 | 3.86E-01 | 2.30E-01 | **3.10E-03** |
| quiescin Q6 isoform a | 1.36 | 1.50 | 3.26 | 2.04 | 2.40 | 4.89 | 5.15E-01 | **2.30E-03** | **2.20E-03** |
| retinoblastoma-associated factor 600 | -1.13 | 1.39 | -1.03 | 1.23 | 1.10 | 1.35 | 2.80E-01 | 1.74E-01 | **3.50E-03** |
| RNA 3&apos;-terminal phosphate cyclase-like protein | -1.07 | -1.33 | 1.95 | -1.41 | 2.08 | 1.47 | **6.20E-03** | 8.64E-02 | 2.58E-02 |
| SH3-binding kinase | -1.27 | 1.92 | -1.22 | 1.51 | 1.04 | 1.57 | 8.92E-02 | 2.36E-01 | **2.00E-04** |
| similar to butyrophilin-like 2 | 1.06 | 1.41 | -2.16 | 1.49 | -2.28 | -1.53 | **8.00E-04** | 6.35E-02 | 3.23E-02 |
| Small inducible cytokine A4 homolog | -1.11 | 1.14 | 1.34 | 1.02 | 1.49 | 1.52 | 3.83E-01 | 9.20E-02 | **2.60E-03** |
| Stathmin | -1.06 | -2.10 | 2.14 | -2.23 | 2.27 | 1.02 | **4.60E-03** | 8.60E-01 | 7.11E-01 |
| T-cell receptor T3 delta chain | 1.21 | 1.04 | 8.51 | 1.25 | 7.05 | 8.82 | **8.00E-04** | **1.00E-04** | **2.00E-04** |
| Trafficking protein particle complex subunit 4 | -1.21 | 1.39 | 1.26 | 1.15 | 1.52 | 1.74 | 8.36E-01 | 1.49E-01 | **4.00E-04** |
| Transposon TX1 hypothetical 149 kDa protein | 1.20 | -1.02 | -2.1 | 1.18 | -2.53 | -2.14 | 3.00E-02 | 3.07E-02 | **3.20E-03** |
| Tubulointersititial nephritis antigen-related protein | -1.04 | -1.22 | 3.85 | -1.27 | 4.00 | 3.15 | 3.38E-02 | 1.45E-02 | **2.80E-03** |
| Type II antifreeze protein | -1.59 | 2.12 | -3.88 | 1.33 | -2.44 | -1.83 | **4.00E-04** | **4.00E-04** | 4.04E-01 |
| Tyrosine aminotransferase | 1.01 | -1.12 | 2.64 | -1.11 | 2.62 | 2.37 | **1.20E-03** | **5.00E-04** | **6.00E-04** |
| UnAn_20939 | 1.08 | 1.52 | -1.69 | 1.65 | -1.83 | -1.11 | **5.80E-03** | 8.89E-01 | 2.81E-01 |
| UnAn_21166 | -1.30 | 1.23 | 1.26 | -1.06 | 1.64 | 1.55 | 3.78E-01 | 3.38E-01 | **1.40E-03** |
| UnAn_21996 | 1.03 | 1.01 | 1.84 | 1.04 | 1.78 | 1.85 | 7.51E-02 | **3.70E-03** | **5.00E-04** |
| UnAn_22121 | -1.25 | -1.46 | -1.38 | -1.82 | -1.11 | -2.02 | 3.31E-01 | **1.90E-03** | 3.55E-02 |
| UnAn_22239 | 1.04 | -1.31 | -1.19 | -1.26 | -1.24 | -1.56 | 8.76E-01 | 7.75E-02 | **3.70E-03** |
| UnAn_22354 | -1.66 | 3.06 | -3.45 | 1.84 | -2.07 | -1.13 | **3.20E-03** | 4.95E-02 | 1.83E-01 |
| UnAn_22442 | 1.11 | 1.03 | -1.28 | 1.14 | -1.43 | -1.25 | 5.18E-02 | 2.18E-01 | **1.00E-03** |
| UnAn_22453 | -1.08 | 1.26 | 1.10 | 1.17 | 1.19 | 1.39 | 7.76E-01 | 7.38E-02 | **3.70E-03** |
| UnAn_22706 | 1.05 | -1.05 | -1.60 | -1.00 | -1.68 | -1.68 | 1.29E-01 | 1.43E-02 | **1.00E-04** |
| UnAn_22816 | -1.18 | -1.24 | -1.23 | -1.47 | -1.04 | -1.53 | 4.81E-01 | **6.10E-03** | 9.20E-02 |
| UnAn_22871 | 1.02 | -1.08 | -2.37 | -1.07 | -2.40 | -2.56 | 1.95E-02 | **1.10E-03** | **7.00E-04** |
| UnAn_22872 | -1.22 | -1.02 | -4.02 | -1.25 | -3.28 | -4.11 | 8.40E-03 | **3.00E-04** | **2.20E-03** |
| UnAn_22873 | -1.54 | 2.60 | -5.08 | 1.69 | -3.30 | -1.96 | **1.00E-04** | **2.00E-04** | 1.32E-01 |
| UnAn_23047 | -1.08 | 1.06 | 1.74 | -1.02 | 1.88 | 1.85 | 1.89E-02 | **5.40E-03** | **1.90E-03** |
| UnAn_23180 | -1.14 | -1.05 | 2.60 | -1.20 | 2.96 | 2.47 | **2.90E-03** | **5.00E-03** | **1.70E-03** |
| UnAn_23258 | 1.19 | -1.51 | -1.92 | -1.27 | -2.28 | -2.90 | 2.49E-01 | 7.80E-03 | **2.10E-03** |
| UnAn_23396 | -1.08 | 1.05 | -2.04 | -1.03 | -1.89 | -1.95 | 1.29E-01 | 1.27E-02 | **6.20E-03** |
| UnAn_23417 | 1.13 | -1.04 | -1.56 | 1.09 | -1.77 | -1.61 | 1.09E-01 | 7.62E-02 | **4.90E-03** |
| UnAn_23426 | -1.09 | 1.28 | 1.27 | 1.17 | 1.39 | 1.62 | 7.82E-01 | 4.80E-02 | **2.50E-03** |
| UnAn_23456 | -1.01 | 1.22 | -3.11 | 1.21 | -3.10 | -2.55 | **2.80E-03** | **1.20E-03** | **7.00E-04** |
| UnAn_23457 | 1.02 | 1.22 | -2.85 | 1.25 | -2.91 | -2.34 | **1.70E-03** | **3.10E-03** | **3.30E-03** |
| UnAn_23472 | -1.02 | 1.25 | -2.98 | 1.22 | -2.93 | -2.39 | 3.63E-02 | 1.25E-02 | **6.00E-04** |
| UnAn_23473 | 1.04 | -1.31 | -2.43 | -1.26 | -2.54 | -3.19 | 3.63E-02 | **4.00E-04** | **3.00E-04** |
| UnAn_23553 | -1.24 | -1.40 | 1.79 | -1.73 | 2.22 | 1.28 | **6.50E-03** | 8.52E-01 | 2.73E-02 |
| UnAn_23610f | -1.00 | -1.27 | 2.53 | -1.27 | 2.54 | 1.99 | **4.10E-03** | **2.70E-03** | **2.00E-04** |
| UnAn_23610r | -1.09 | 1.44 | 3.43 | 1.32 | 3.74 | 4.93 | 8.97E-02 | **1.00E-03** | **4.30E-05** |
| UnAn_23738 | -1.00 | 1.28 | -3.48 | 1.27 | -3.46 | -2.72 | 8.60E-03 | **4.30E-03** | **1.70E-03** |
| UnAn_26838 | 1.15 | -1.48 | -1.47 | -1.29 | -1.69 | -2.18 | 5.80E-01 | **2.30E-03** | **4.30E-05** |
| UnAn_26842 | -1.24 | 1.00 | 1.10 | -1.24 | 1.37 | 1.10 | 9.18E-02 | 2.21E-01 | **2.40E-03** |
| UnAn_26954 | 1.05 | -1.44 | -1.12 | -1.38 | -1.17 | -1.62 | 4.99E-01 | 3.52E-02 | **4.10E-03** |
| UnAn_27136 | -1.09 | -1.04 | -1.35 | -1.14 | -1.24 | -1.41 | 2.57E-01 | **2.40E-03** | 1.90E-02 |
| UnAn_27220 | 1.09 | 1.12 | -2.79 | 1.22 | -3.04 | -2.50 | **3.00E-03** | **4.60E-03** | **2.60E-03** |
| UnAn_27282 | -1.05 | 2.14 | -2.78 | 2.05 | -2.65 | -1.29 | **2.60E-03** | 2.18E-01 | 3.48E-01 |
| UnAn_27313 | 1.09 | 1.17 | -1.26 | 1.28 | -1.38 | -1.08 | 6.73E-02 | 8.91E-01 | **2.40E-03** |
| UnAn_27329 | 1.03 | -1.02 | -1.49 | 1.01 | -1.53 | -1.52 | 8.25E-02 | 1.37E-02 | **2.60E-03** |
| UnAn_27580 | -1.10 | -1.11 | -1.54 | -1.22 | -1.40 | -1.71 | 3.15E-01 | **2.60E-03** | 7.90E-03 |
| UnAn_27682 | -1.02 | -1.32 | -1.18 | -1.35 | -1.16 | -1.57 | 6.28E-01 | 1.64E-02 | **4.00E-03** |
| UnAn_27774 | -1.04 | -1.54 | -1.25 | -1.61 | -1.20 | -1.93 | 4.62E-01 | 8.70E-03 | **6.50E-03** |
| UnAn_27892 | -1.26 | -1.22 | 1.54 | -1.54 | 1.95 | 1.27 | 2.16E-02 | 9.77E-01 | **5.90E-03** |
| UnAn_27910 | 1.14 | -1.25 | 6.24 | -1.10 | 5.49 | 4.98 | **5.00E-05** | **1.40E-05** | **1.00E-04** |
| UnAn_27931 | -1.45 | -1.44 | -1.27 | -2.09 | 1.15 | -1.82 | 8.06E-02 | **7.00E-04** | 1.83E-01 |
| UnAn_27936 | 1.03 | -1.73 | 2.00 | -1.68 | 1.95 | 1.16 | **2.60E-03** | 2.76E-01 | 3.81E-01 |
| UnAn_27946 | -1.01 | 1.06 | -1.64 | 1.05 | -1.62 | -1.54 | 4.28E-02 | 9.20E-03 | **1.70E-03** |
| UnAn_27991 | -1.05 | -1.20 | -1.58 | -1.26 | -1.51 | -1.90 | 3.70E-01 | **5.20E-03** | 1.19E-02 |
| UnAn_28041 | -1.13 | -1.09 | 3.78 | -1.23 | 4.26 | 3.45 | **5.30E-03** | **1.80E-03** | **3.00E-04** |
| UnAn_28072 | 1.38 | -1.21 | 1.71 | 1.14 | 1.24 | 1.41 | 1.80E-01 | **4.20E-03** | 8.50E-01 |
| UnAn_28168 | 1.02 | -1.28 | 1.05 | -1.25 | 1.02 | -1.23 | 4.73E-02 | 3.02E-02 | **4.90E-03** |
| UnAn_28216 | -1.11 | 1.15 | -2.06 | 1.04 | -1.85 | -1.79 | 2.23E-02 | **4.40E-03** | 1.87E-02 |
| UnAn_28350 | 1.16 | -1.28 | -1.39 | -1.10 | -1.61 | -1.78 | 4.16E-01 | 4.12E-02 | **3.70E-03** |
| UnAn_28436 | -1.05 | 1.77 | -1.85 | 1.68 | -1.76 | -1.05 | **2.00E-03** | 4.95E-01 | 9.75E-01 |
| UnAn_28575 | 1.09 | 1.36 | -1.52 | 1.48 | -1.65 | -1.12 | **2.50E-03** | 8.34E-01 | 2.30E-01 |
| UnAn_28749 | 1.10 | 1.96 | -2.83 | 2.16 | -3.12 | -1.44 | **7.00E-04** | 2.36E-01 | 7.99E-02 |
| UnAn_28776 | 1.16 | 1.06 | 2.14 | 1.23 | 1.84 | 2.26 | 1.82E-01 | **3.30E-03** | **4.60E-03** |
| UnAn_28943 | 1.10 | 1.21 | -1.80 | 1.33 | -1.98 | -1.49 | 1.74E-02 | 7.60E-02 | **3.00E-04** |
| UnAn_29008 | 1.03 | 1.18 | 1.95 | 1.22 | 1.90 | 2.31 | 1.57E-02 | **9.00E-04** | **1.20E-03** |
| UnAn_29009 | -1.11 | -1.30 | 2.96 | -1.45 | 3.30 | 2.27 | 1.91E-02 | 3.12E-02 | **2.20E-03** |
| UnAn_29159 | 1.09 | -1.13 | 5.14 | -1.04 | 4.73 | 4.53 | **2.20E-03** | **2.00E-04** | **3.00E-04** |
| UnAn_29186 | -1.27 | -1.18 | -4.11 | -1.51 | -3.23 | -4.86 | 6.84E-02 | **4.20E-03** | 2.00E-02 |
| UnAn_29337 | 1.06 | -1.51 | -1.09 | -1.42 | -1.16 | -1.65 | 3.69E-01 | 3.18E-02 | **4.80E-03** |
| UnAn_29411 | -1.04 | -1.05 | 12.88 | -1.09 | 13.42 | 12.3 | **2.00E-04** | **1.00E-04** | **1.00E-04** |
| UnAn_29519 | 1.30 | -1.51 | -1.24 | -1.16 | -1.61 | -1.87 | 8.73E-01 | 1.77E-01 | **4.00E-03** |
| UnAn_29527 | 1.06 | -1.26 | -1.11 | -1.19 | -1.17 | -1.40 | 7.61E-01 | 7.63E-02 | **5.60E-03** |
| UnAn_29734 | 1.23 | 1.21 | -2.18 | 1.49 | -2.68 | -1.80 | **1.20E-03** | 3.16E-02 | **1.20E-03** |
| UnAn_29811 | 1.10 | -1.41 | -1.03 | -1.29 | -1.13 | -1.45 | 3.88E-01 | 6.22E-02 | **1.00E-04** |
| UnAn_29863 | 1.16 | 1.23 | -1.54 | 1.43 | -1.78 | -1.25 | 7.69E-02 | 6.97E-01 | **9.00E-04** |
| Uridine phosphorylase 1 | -1.06 | -1.25 | 3.64 | -1.32 | 3.85 | 2.91 | **9.00E-04** | **1.00E-03** | **5.00E-04** |
| VAMP-associated protein A | 1.37 | -1.09 | 1.05 | 1.25 | -1.31 | -1.04 | 4.82E-01 | 7.78E-02 | **2.70E-03** |
| Zinc finger protein 330 | -1.01 | 1.18 | -2.45 | 1.17 | -2.42 | -2.07 | 5.79E-02 | 2.48E-02 | **5.80E-03** |
| Zinc finger protein HRX | -1.11 | 1.13 | 1.27 | 1.02 | 1.42 | 1.44 | 3.81E-01 | 9.91E-02 | **2.50E-03** |
