# Supplementary material for: Transcriptomic assessment of resistance to effects of an aryl hydrocarbon receptor (AHR) agonist in embryos of Atlantic killifish (Fundulus heteroclitus) from a marine Superfund site
Source: BMC Genomics. 2011 May 24;12:263. doi: 10.1186/1471-2164-12-263 (PMC3213123; doi:10.1186/1471-2164-12-263)
Supplement: Additional file 4 — Table S2. Differential gene expression and PCB inducibility in pairwise comparisons of NBH and SC embryos at 5, 10, and 15 dpf. Genes with significant differences in pairwise comparisons of gene expression are included. Gene expression ratios are indicated. A gene with a positive fold-difference is more highly expressed in the population/treatment listed first, and a gene with a negative fold-difference is more highly expressed in the population/treatment listed last. Genes are listed in order of ratios in the reference population (SC) comparison with tolerant population (SC). Ratios with significant p-values are in bold. See Table S1 (Additional file 2) for a list of all genes significant in the ANOVA analysis. NBH: New Bedford Harbor; SC: Scorton Creek; PCB: PCB-126; DMSO: dimethylsulfoxide. Unannotated genes are denoted by UnAn and a unique number. Some of the unannotated probes were subsequently annotated after extension using the 454 database; see Table S4 (Additional file 6) for details. [file 1471-2164-12-263-S4.DOC]

Oleksiak et al. BMC Genomics

**Additional file 4: Table S2. Differential gene expression and PCB inducibility in pairwise comparisons of NBH and SC embryos at 5, 10, and 15 dpf.**

| **Gene** | **NBH PCB / NBH DMSO** | **SC PCB / SC DMSO** | **NBH DMSO / SC DMSO** |
| --- | --- | --- | --- |
| **5 days post fertilization** |  |  |  |
| basic leucine zipper nuclear factor 1 [Mus musculus]_603 | -1.03 | **5.09**^c^ | -1.52 |
| Cytochrome P450 1A1 (EC 1.14.14.1) (CYPIA1)_1115 | 1.29 | **4.82**^b,c^ | -1.12 |
| *UnAn_29411_6378 (CYP1B1) | -1.03 | **4.61**^b,c^ | -2.51 |
| T-cell surface glycoprotein CD3 delta chain precursor (T-cell receptor T3 delta chain)_3770 | 1.14 | **2.92**^b,c^ | -1.64 |
| *UnAn_27910_5543 (CYP1B1) | 1.22 | **2.54**^b,c^ | -1.32 |
| Diablo homolog, mitochondrial precursor (Second mitochondria-derived activator of caspase) (Smac protein) (Direct IAP binding protein with low pI)_1191 | -1.03 | **2.40** | 2.14 |
| UnAn_21996_4295 | 1.28 | **1.84**^b,c^ | 1.02 |
| Small inducible cytokine A4 homolog precursor (Macrophage inflammatory protein 1-beta homolog)_3581 | 1.13 | **1.78**^c^ | 1.03 |
| Synaptophysin-like protein (Pantophysin)_3753 | -1.15 | **1.43** | 1.64 |
| ATP synthase lipid-binding protein, mitochondrial precursor (EC 3.6.3.14) (ATP synthase proteolipid P2) (ATPase protein 9) (ATPase subunit C)_560 | **1.79** | 1.31 | 1.47 |
| Hypothetical protein C31G5.21 in chromosome I_1963 | 1.12 | 1.27 | **1.98** |
| hect domain and RLD 4 isoform b [Homo sapiens]_1806 | **1.33** | 1.21 | -1.06 |
| UnAn_26723_4896 | -1.14 | 1.14 | **2.12** |
| UnAn_23246_4614 | -1.09 | 1.03 | **1.96** |
| UnAn_22726_4401 | 1.03 | -1.10 | **1.88** |
| Proteasome subunit beta type 7 precursor (EC 3.4.25.1) (Proteasome subunit Z) (Macropain chain Z) (Multicatalytic endopeptidase complex chain Z)_3109 | **1.41** | -1.10 | -1.14 |
| Deoxyribonuclease-1 precursor (EC 3.1.21.1) (Deoxyribonuclease I) (DNase I)_1181 | 1.48 | -1.14 | **-2.57** |
| Guanidinoacetate N-methyltransferase (EC 2.1.1.2)_1754 | -1.16 | -1.25 | **-3.28** |
|  |  |  |  |
| **10 days post fertilization** |  |  |  |
| *UnAn_29411_6378 (CYP1B1) | 1.09 | **11.78**^a,c^ | 1.12 |
| Cytochrome P450 1A1 (EC 1.14.14.1) (CYPIA1)_1115 | 1.10 | **6.10**^a,c^ | -1.13 |
| T-cell surface glycoprotein CD3 delta chain precursor (T-cell receptor T3 delta chain)_3770 | 1.07 | **5.47**^a,c^ | -1.25 |
| *UnAn_27910_5543 (CYP1B1) | -1.02 | **3.36**^a,c^ | -1.29 |
| Tubulointerstitial nephritis antigen-like precursor (Androgen-regulated gene protein 1) (Adrenocortical zonation factor 1) (AZ-1) (Tubulointersititial nephritis antigen-related protein) (TARP)_4042 | 1.49 | **3.00**^c^ | -1.03 |
| **UnAn_29159_6257 (apolipoprotein E) | 1.18 | **2.66**^c^ | -1.20 |
| **UnAn_28041_5632 (apolipoprotein E) | 1.02 | **2.43**^c^ | -1.01 |
| Acidic phosphoprotein precursor (50 kDa antigen)_277 | 1.11 | **2.37**^c^ | -1.15 |
| **UnAn_23610r_4780 (apolipoprotein E) | 1.06 | **2.30**^c^ | -1.45 |
| UnAn_21996_4295 | -1.15 | **2.09**^a,c^ | -1.01 |
| Apomucin (Mucin core protein) (Fragment)_492 | 1.16 | **2.02**^c^ | 1.45 |
| C61 protein [Mus musculus]_684 | 1.22 | **1.99**^c^ | 1.84 |
| WAP four-disulfide core domain protein 3 precursor (Putative protease inhibitor WAP14)_6790 | 1.02 | **1.96** | 1.59 |
| Tyrosine aminotransferase (EC 2.6.1.5) (L-tyrosine:2-oxoglutarate aminotransferase) (TAT)_4064 | -1.14 | **1.90**^c^ | -1.24 |
| MHC class II transactivator (CIITA)_2363 | 1.03 | **1.76**^c^ | -1.06 |
| UnAn_23121_4564 | -1.09 | **1.75** | 1.54 |
| Troponin C, slow skeletal and cardiac muscles (TN-C)_3995 | 1.08 | 1.63 | **2.68** |
| Epsin-4 (Epsin-related protein) (EpsinR) (Enthoprotin)_1384 | 1.38 | **1.62** | 2.03 |
| UnAn_27985_5592 | -1.10 | **1.44** | 1.13 |
| AMP deaminase 1 (EC 3.5.4.6) (Myoadenylate deaminase) (AMP deaminase isoform M)_431 | 1.27 | **1.43** | 1.49 |
| UnAn_29343_6349 | 1.20 | **1.31** | 1.48 |
| UnAn_29849_6655 | 1.11 | 1.24 | **1.67** |
| Cathepsin D precursor (EC 3.4.23.5)_755 | **-1.45** | 1.04 | 1.01 |
| UnAn_22785_4432 | -1.24 | -1.01 | **-1.84** |
| UnAn_20648_4152 | **1.32** | -1.05 | 1.01 |
| Mitogen-activated protein kinase kinase 1 interacting protein 1 (MEK binding partner 1) (Mp1)_2424 | -1.10 | -1.07 | **-1.51** |
| Epididymal secretory glutathione peroxidase precursor (EC 1.11.1.9) (Epididymis-specific glutathione peroxidase-like protein) (EGLP)_1378 | **-1.73** | -1.15 | -1.32 |
| Muted protein homolog_2461 | **-1.60** | -1.16 | -1.50 |
| UnAn_22879_4456 | **1.56** | -1.20 | -1.51 |
| UnAn_22354_4332 | -1.09 | -1.28 | **2.13**^c^ |
| Type II antifreeze protein precursor (AFP)_4059 | -1.12 | -1.30 | **1.72**^c^ |
| T-cell surface glycoprotein CD8 beta chain precursor (CD8 antigen 37 kDa chain) (OX-8 membrane antigen)_3771 | **1.51** | -1.32 | -1.12 |
| UnAn_20957_4180 | **-1.84** | -1.32 | -1.48 |
| Chromobox protein homolog 2 (Modifier 3 protein) (M33)_827 | **-1.65** | -1.47 | -1.36 |
| Beta enolase (EC 4.2.1.11) (2-phospho-D-glycerate hydro-lyase) (Muscle-specific enolase) (MSE) (Skeletal muscle enolase) (Enolase 3)_620 | -1.00 | **-1.53** | -1.29 |
| UnAn_27466_5284 | 1.30 | **-1.54** | -1.33 |
| Transcription factor PU.1_3890 | 1.19 | **-1.55** | -1.36 |
| UnAn_22873_4452 | -1.00 | **-1.55**^c^ | 1.30 |
| RWD domain containing protein 1 (Small androgen receptor-interacting protein)_3458 | 1.05 | **-1.73** | -1.05 |
| Parvalbumin beta_2778 | 1.30 | **-1.80**^c^ | -1.49 |
|  |  |  |  |
| **15 days post fertilization** |  |  |  |
| *UnAn_29411_6378 (CYP1B1) | -1.04 | **12.30**^a,b^ | -1.05 |
| Cytochrome P450 1A1 (EC 1.14.14.1) (CYPIA1)_1115 | -1.19 | **10.27**^a,b^ | 1.17 |
| T-cell surface glycoprotein CD3 delta chain precursor (T-cell receptor T3 delta chain)_3770 | 1.21 | **8.82**^a,b^ | 1.04 |
| *UnAn_27910_5543 (CYP1B1) | 1.14 | **4.98**^a,b^ | -1.25 |
| **UnAn_23610r_4780 (apolipoprotein E) | -1.09 | **4.93**^b^ | 1.44 |
| quiescin Q6 isoform a [Homo sapiens]_3294 | 1.36 | **4.89** | 1.50 |
| **UnAn_29159_6257 (apolipoprotein E) | 1.09 | **4.53**^b^ | -1.13 |
| Acidic phosphoprotein precursor (50 kDa antigen)_277 | -1.09 | **4.40**^b^ | -1.09 |
| basic leucine zipper nuclear factor 1 [Mus musculus]_603 | 1.30 | **3.60**^a^ | 1.17 |
| **UnAn_28041_5632 (apolipoprotein E) | -1.13 | **3.45**^b^ | -1.09 |
| Probable pancreatic secretory proteinase inhibitor (PSTI type)_3040 | -1.03 | **3.29** | 1.39 |
| Tubulointerstitial nephritis antigen-like precursor (Androgen-regulated gene protein 1) (Adrenocortical zonation factor 1) (AZ-1) (Tubulointersititial nephritis antigen-related protein) (TARP)_4042 | -1.04 | **3.15**^b^ | -1.22 |
| Uridine phosphorylase 1 (EC 2.4.2.3) (UrdPase 1) (UPase 1)_6714 | -1.06 | **2.91** | -1.25 |
| Atrial natriuteric peptide-converting enzyme (EC 3.4.21.-) (pro-ANP-converting enzyme) (Corin) (Heart specific serine proteinase ATC2) (Transmembrane protease, serine 10)_581 | 1.13 | **2.82** | -1.24 |
| Hypothetical 26.3 kDa protein in RAD4-CHD1 intergenic region_1951 | 1.14 | **2.72** | -1.11 |
| UnAn_23180_4586 | -1.14 | **2.47** | -1.05 |
| Tyrosine aminotransferase (EC 2.6.1.5) (L-tyrosine:2-oxoglutarate aminotransferase) (TAT)_4064 | 1.01 | **2.37**^b^ | -1.12 |
| UnAn_29008_6183 | 1.03 | **2.31** | 1.18 |
| Protein C18orf37 homolog_3139 | 1.10 | **2.28** | -1.12 |
| UnAn_29009_6184 | -1.11 | **2.27** | -1.30 |
| UnAn_28776_6045 | 1.16 | **2.26** | 1.06 |
| Ependymin precursor (EPD)_1371 | -1.07 | **2.25** | 1.13 |
| Catalase (EC 1.11.1.6)_747 | 1.13 | **2.20** | -1.11 |
| ETHE1 protein [Mus musculus]_1408 | -1.29 | **2.17** | -1.03 |
| Proteasome subunit alpha type 2 (EC 3.4.25.1) (Proteasome component C3) (Macropain subunit C3) (Multicatalytic endopeptidase complex subunit C3) (XC3)_3093 | 1.33 | **2.17** | 1.31 |
| NDRG1 protein (N-myc downstream regulated gene 1 protein) (Protein Ndr1)_2613 | 1.03 | **2.07** | 1.56 |
| Lymphocyte antigen Ly-6D precursor (E48 antigen)_2288 | -1.19 | **2.01** | 1.49 |
| UnAn_23610f_4779 | -1.00 | **1.99** | -1.27 |
| Catalase (EC 1.11.1.6)_746 | 1.34 | **1.91** | -1.00 |
| Isocitrate dehydrogenase [NADP] cytoplasmic (EC 1.1.1.42) (Oxalosuccinate decarboxylase) (IDH) (NADP(+)-specific ICDH) (IDP)_2174 | -1.18 | **1.91** | 1.69 |
| Desmin_1185 | 1.11 | **1.88** | 1.15 |
| UnAn_21996_4295 | 1.03 | **1.85**^a,b^ | 1.01 |
| UnAn_23047_4537 | -1.08 | **1.85** | 1.06 |
| hypothetical protein LOC76747 [Mus musculus]_2043 | -1.07 | **1.83** | 1.03 |
| Angiogenic factor with G patch and FHA domains 1 (Angiogenic factor VG5Q) (Vasculogenesis gene on 5q) (hVG5Q)_437 | -1.04 | **1.80** | 1.16 |
| Trafficking protein particle complex subunit 4 (Synbindin) (TRS23 homolog)_3861 | -1.21 | **1.74** | 1.39 |
| Prostaglandin E2 receptor, EP4 subtype (Prostanoid EP4 receptor) (PGE receptor, EP4 subtype)_3080 | -1.30 | **1.71** | -1.00 |
| BTG1 protein (B-cell translocation gene 1 protein)_680 | 1.04 | **1.70** | -1.27 |
| Homogentisate 1,2-dioxygenase (EC 1.13.11.5) (Homogentisicase) (Homogentisate oxygenase) (Homogentisic acid oxidase)_1922 | 1.12 | **1.69** | 1.05 |
| C61 protein [Mus musculus]_684 | -1.01 | **1.67**^b^ | 1.12 |
| Ferritin, middle subunit (EC 1.16.3.1) (Ferritin M)_1504 | -1.30 | **1.64** | 1.13 |
| Microsomal glutathione S-transferase 3 (EC 2.5.1.18) (Microsomal GST-3) (Microsomal GST-III)_2370 | 1.05 | **1.63** | 1.37 |
| UnAn_23426_4694 | -1.09 | **1.62** | 1.28 |
| CXXC finger 5 [Mus musculus]_1022 | -1.05 | **1.62** | -1.11 |
| L-lactate dehydrogenase B chain (EC 1.1.1.27) (LDH-B)_2272 | 1.22 | **1.61** | -1.08 |
| Complement factor H-related protein 1 precursor (FHR-1) (H factor-like protein 1) (H-factor-like 1) (H36)_965 | 1.05 | **1.60** | 1.05 |
| MHC class II transactivator (CIITA)_2363 | 1.16 | **1.60**^b^ | -1.07 |
| SH3-binding kinase [Rattus norvegicus]_3551 | -1.27 | **1.57** | **1.92** |
| Death-associated protein 1 (DAP-1)_1164 | 1.15 | **1.56** | -1.02 |
| UnAn_21166_4205 | -1.30 | **1.55** | 1.23 |
| Small inducible cytokine A4 homolog precursor (Macrophage inflammatory protein 1-beta homolog)_3581 | -1.11 | **1.52**^a^ | 1.14 |
| Potassium/sodium hyperpolarization-activated cyclic nucleotide-gated channel 2 (Brain cyclic nucleotide gated channel 2) (BCNG-2)_2946 | -1.10 | **1.51** | 1.22 |
| Dual specificity protein kinase CLK1 (EC 2.7.1.37) (EC 2.7.1.112) (CDC-like kinase 1)_1275 | -1.05 | **1.45** | 1.12 |
| Zinc finger protein HRX (ALL-1) (Trithorax-like protein)_6862 | -1.11 | **1.44** | 1.13 |
| UnAn_22453_4351 | -1.08 | **1.39** | 1.26 |
| Major vault protein (MVP) (Fragment)_2313 | -1.24 | **1.36** | 1.16 |
| Protein C20orf149_3143 | -1.01 | **1.36** | -1.02 |
| retinoblastoma-associated factor 600 [Homo sapiens]_3364 | -1.13 | **1.35** | 1.39 |
| Magnesium-chelatase subunit chlI (EC 6.6.1.1) (Mg-protoporphyrin IX chelatase)_2310 | 1.06 | **1.34** | -1.06 |
| Nuclear protein 1 (Protein p8) (Candidate of metastasis 1)_2690 | **1.30** | 1.26 | -1.15 |
| Deoxyribonuclease II alpha precursor (EC 3.1.22.1) (DNase II alpha) (Acid DNase) (Lysosomal DNase II) (R31240_2)_1179 | 1.08 | **1.25** | 1.06 |
| UnAn_26842_4966 | **-1.24** | 1.10 | 1.00 |
| lymphocyte specific 1 [Mus musculus]_2290 | 1.07 | 1.09 | **1.55** |
| Cytochrome P450 2J5 (EC 1.14.14.1) (CYPIIJ5) (Arachidonic acid epoxygenase)_1128 | **-1.31** | -1.01 | -1.09 |
| Vesicle-associated membrane protein-associated protein A (VAMP-associated protein A) (VAMP-A) (VAP-A) (33 kDa Vamp-associated protein) (VAP-33)_6752 | **1.37** | -1.04 | -1.09 |
| UnAn_28436_5873 | -1.05 | -1.05 | **1.77** |
| UnAn_22354_4332 | -1.66 | -1.13 | **3.06**^b^ |
| UnAn_28168_5719 | 1.02 | **-1.23** | **-1.28** |
| UnAn_22442_4346 | 1.11 | **-1.25** | 1.03 |
| Putative deoxyribose-phosphate aldolase (EC 4.1.2.4) (Phosphodeoxyriboaldolase) (Deoxyriboaldolase) (DERA)_3257 | 1.11 | **-1.31** | -1.07 |
| Protein disulfide-isomerase A4 precursor (EC 5.3.4.1) (Protein ERp-72) (ERp72)_3171 | 1.20 | **-1.38** | -1.20 |
| UnAn_29527_6431 | 1.06 | **-1.40** | -1.26 |
| UnAn_27136_5101 | -1.09 | **-1.41** | -1.04 |
| 30S ribosomal protein S12_57 | 1.02 | **-1.45** | 1.14 |
| UnAn_29811_6629 | 1.10 | **-1.45** | -1.41 |
| UnAn_28943_6156 | 1.10 | **-1.49** | 1.21 |
| UnAn_27329_5204 | 1.03 | **-1.52** | -1.02 |
| UnAn_22816_4445 | -1.18 | **-1.53** | -1.24 |
| UnAn_27946_5561 | -1.01 | **-1.54** | 1.06 |
| Aminoacylase-1 (EC 3.5.1.14) (N-acyl-L-amino-acid amidohydrolase) (ACY-1)_429 | -1.19 | **-1.55** | -1.28 |
| hypothetical protein LOC196463 [Homo sapiens]_1986 | -1.05 | **-1.56** | -1.10 |
| Complement component C8 beta chain precursor (Complement component 8 beta subunit)_949 | **-1.37** | **-1.56** | -1.14 |
| UnAn_22239_4326 | 1.04 | **-1.56** | -1.31 |
| UnAn_27682_5419 | -1.02 | **-1.57** | -1.32 |
| UnAn_23417_4689 | 1.13 | **-1.61** | -1.04 |
| UnAn_26954_5016 | 1.05 | **-1.62** | -1.44 |
| UnAn_29337_6346 | 1.06 | **-1.65** | -1.51 |
| Brain mitochondrial carrier protein-1 (BMCP-1) (Mitochondrial uncoupling protein 5) (UCP 5) (Solute carrier family 25, member 14)_659 | -1.11 | **-1.67** | 1.10 |
| UnAn_22706_4390 | 1.05 | **-1.68** | -1.05 |
| Guanine nucleotide-binding protein G(T) gamma-T1 subunit (Transducin gamma chain)_1765 | 1.19 | **-1.68** | 1.10 |
| Keratin, type I cytoskeletal 50 kDa (GK50)_2201 | -1.40 | **-1.71** | -1.21 |
| UnAn_27580_5360 | -1.10 | **-1.71** | -1.11 |
| UnAn_28350_5828 | 1.16 | **-1.78** | -1.28 |
| Pre-mRNA branch site protein p14 (SF3B 14 kDa subunit)_3005 | 1.12 | **-1.78** | 1.12 |
| ADP/ATP translocase 3 (Adenine nucleotide translocator 2) (ANT 3) (ADP,ATP carrier protein 3) (Solute carrier family 25, member 6) (ADP,ATP carrier protein, isoform T2) (ANT 2)_356 | -1.03 | **-1.78** | -1.02 |
| UnAn_28216_5745 | -1.11 | **-1.79** | 1.15 |
| UnAn_29734_6576 | 1.23 | **-1.80** | 1.21 |
| UnAn_27931_5556 | -1.45 | **-1.82** | -1.44 |
| Type II antifreeze protein precursor (AFP)_4059 | **-1.59** | **-1.83** | **2.12**^b^ |
| UnAn_29519_6426 | 1.30 | **-1.87** | -1.51 |
| Acidic phosphoprotein precursor (50 kDa antigen)_281 | 1.13 | **-1.90** | -1.11 |
| UnAn_27991_5597 | -1.05 | **-1.90** | -1.20 |
| Plasma serine protease inhibitor precursor (PCI) (Protein C inhibitor) (Plasminogen activator inhibitor-3) (PAI3) (Acrosomal serine protease inhibitor)_2905 | 1.09 | **-1.91** | 1.02 |
| UnAn_27774_5467 | -1.04 | **-1.93** | -1.54 |
| UnAn_23396_4676 | -1.08 | **-1.95** | 1.05 |
| UnAn_22873_4452 | **-1.54** | **-1.96**^b^ | **2.60** |
| Collagen alpha 2(I) chain precursor_907 | 1.24 | **-1.96** | 1.14 |
| Cathepsin Z precursor (EC 3.4.22.-)_767 | 1.12 | **-2.00** | 1.04 |
| Myosin-binding protein C, cardiac-type (Cardiac MyBP-C) (C-protein, cardiac muscle isoform)_2505 | 1.26 | **-2.02** | -1.18 |
| UnAn_22121_4311 | -1.25 | **-2.02** | -1.46 |
| Zinc finger protein 330 (Nucleolar autoantigen 36)_6850 | -1.01 | **-2.07** | 1.18 |
| Transposon TX1 hypothetical 149 kDa protein (ORF 2)_3957 | 1.20 | **-2.14** | -1.02 |
| UnAn_26838_4964 | 1.15 | **-2.18** | -1.48 |
| UnAn_23457_4702 | 1.02 | **-2.34** | 1.22 |
| UnAn_23472_4711 | -1.02 | **-2.39** | 1.25 |
| UnAn_27220_5147 | 1.09 | **-2.50** | 1.12 |
| UnAn_23456_4701 | -1.01 | **-2.55** | 1.22 |
| UnAn_22871_4450 | 1.02 | **-2.56** | -1.08 |
| Collagen alpha 1(X) chain precursor_902 | -1.12 | **-2.59** | 1.31 |
| Parvalbumin beta_2775 | -1.22 | **-2.71** | 1.04 |
| UnAn_23738_4838 | -1.00 | **-2.72** | 1.28 |
| UnAn_23258_4621 | 1.19 | **-2.90** | -1.51 |
| Apomucin (Mucin core protein) (Fragment)_492 | 1.07 | **-2.96**^b^ | -1.70 |
| UnAn_23473_4712 | 1.04 | **-3.19** | -1.31 |
| Parvalbumin beta_2776 | 1.08 | **-3.72** | 1.19 |
| Parvalbumin beta_2778 | -1.02 | **-3.90**^b^ | -1.10 |
| UnAn_22872_4451 | -1.22 | **-4.11** | -1.02 |
| UnAn_29186_6274 | -1.27 | **-4.86** | -1.18 |

Genes with significant differences in pairwise comparisons of gene expression are included. Gene expression ratios are indicated. A gene with a positive fold-difference is more highly expressed in the population/treatment listed first, and a gene with a negative fold-difference is more highly expressed in the population/treatment listed last. Genes are listed in order of ratios in the reference population (SC) comparison with tolerant population (SC). Ratios with significant p-values are in **bold**. See Table S1 (Additional file 2) for a list of all genes significant in the ANOVA analysis. NBH: New Bedford Harbor; SC: Scorton Creek; PCB: PCB-126; DMSO: dimethylsulfoxide. Unannotated genes are denoted by UnAn and a unique number. Some of the unannotated probes were subsequently annotated after extension using the 454 database; see Table S4 (Additional file 6) for details.

*These two probes represent the same transcript, which has been annotated as cytochrome P450 1B1 (CYP1B1) using data from 454 libraries.

** These three probes represent the same transcript, which has been annotated as apolipoprotein E using data from 454 libraries.

^a^Also differentially expressed at 5 dpf.

^b^Also differentially expressed at 10 dpf.

^c^Also differentially expressed at 15 dpf.
