# Supplementary material for: Transcriptomic assessment of resistance to effects of an aryl hydrocarbon receptor (AHR) agonist in embryos of Atlantic killifish (Fundulus heteroclitus) from a marine Superfund site
Source: BMC Genomics. 2011 May 24;12:263. doi: 10.1186/1471-2164-12-263 (PMC3213123; doi:10.1186/1471-2164-12-263)
Supplement: Additional file 5 — Table S3. Mean fold change in gene expression in pairwise comparisons. [file 1471-2164-12-263-S5.DOC]

Oleksiak et al. BMC Genomics

**Additional file 5: Table S3. Mean fold change in gene expression in pairwise comparisons.**
Data are presented as mean ± SD.

|  | **5 dpf** | **10 dpf** | **15 dpf** |
| --- | --- | --- | --- |
| **NBH PCB vs NBH DMSO** | 1.51 ± 0.25 | 1.58 ± 1.16 | 1.39 ± 0.13 |
| **SC PCB vs SC DMSO** | 3.05 ± 1.42 | 2.63 ± 2.19 | 2.33 ± 1.56 |
| **NBH DMSO vs SC DMSO** | 2.30 ± 0.54 | 1.93 ± 0.42 | 2.04 ± 0.61 |
