# Supplementary material for: Transcriptomic assessment of resistance to effects of an aryl hydrocarbon receptor (AHR) agonist in embryos of Atlantic killifish (Fundulus heteroclitus) from a marine Superfund site
Source: BMC Genomics. 2011 May 24;12:263. doi: 10.1186/1471-2164-12-263 (PMC3213123; doi:10.1186/1471-2164-12-263)
Supplement: Additional file 6 — Table S4. Probes annotated as a result of 454 sequencing. Microarray probe sequences were used in blast searches against the 454 sequence data (EST and shotgun libraries). The probe sequences that were extended with the matching reads were used to search GenBank using blast to obtain the annotations. [file 1471-2164-12-263-S6.DOC]

Oleksiak et al. BMC Genomics

**Additional file 6: Table S4. Probes annotated as a result of 454 sequencing.**

Microarray probe sequences were used in blast searches against the 454 sequence data (EST and shotgun libraries). The probe sequences that were extended with the matching reads were used to search GenBank using blast to obtain the annotations.

| **Probe Name** | **Annotation** | **e-value** |
| --- | --- | --- |
| UnAn_20648_4152 | C-type lectin [Fundulus heteroclitus] AAU50548 | 2e-14 |
| UnAn_22879_4456 | eukaryotic translation initiation factor 1B [Danio rerio] NP_955882 | 4e-47 |
| UnAn_23121_4564 | male-specific protein [Sarotherodon galilaeus] AAR19269 | 1e-48 |
| UnAn_23610r_4780  UnAn_28041_5632  UnAn_29159_6257 | Apolipoprotein E [Oplegnathus fasciatus] ACF21982 | 5e-85 |
| UnAn_29343_6349 | High mobility group protein B2 [Anoplopoma fimbria] ACQ58903 | 2e-14 |
| UnAn_29849_6655 | Galectin-3-binding protein [Salmo salar] NP_001135263 | 1e-22 |
| UnAn_27910_5543  UnAn_29411_6378 | Cytochrome P450 family 1 subfamily B polypeptide 1 [Fundulus heteroclitus] FJ786959 | 2e-13 |
| UnAn_22726_4401 | Transmembrane protein 66 [Salmo salar] NP_001133668 | 6e-04 |
