# Supplementary material for: Transcriptomic assessment of resistance to effects of an aryl hydrocarbon receptor (AHR) agonist in embryos of Atlantic killifish (Fundulus heteroclitus) from a marine Superfund site
Source: BMC Genomics. 2011 May 24;12:263. doi: 10.1186/1471-2164-12-263 (PMC3213123; doi:10.1186/1471-2164-12-263)
Supplement: Additional file 7 — Figure S3. Possible scenarios comparing the response of SC and NBH fish to PCB. [file 1471-2164-12-263-S7.PDF]

Figure S3

Possible results of an array study of SC and NBH fish

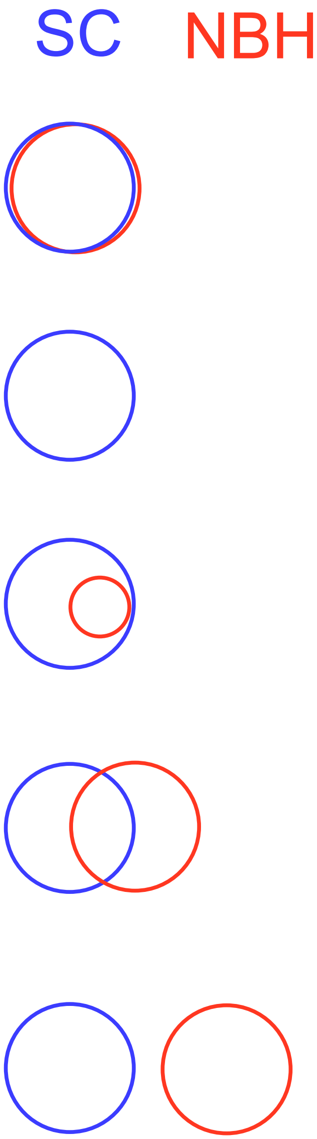

a) Complete overlap - no difference in PCB-altered gene expression

b) NBH fish completely refractory to PCB-altered gene expression

c) Some but not all genes that respond to PCB in SC fish
are responsive in NBH fish

d) Partial overlap - overlapping but distinct PCB-altered gene sets

e) No overlap - completely distinct PCB-altered gene sets
